# Adaptive dynamics of diverging fitness optima

**DOI:** 10.1007/s00285-025-02270-5

**Published:** 2025-10-13

**Authors:** Manh Hong Duong, Fabian Spill, Blaine Van Rensburg

**Affiliations:** School of Mathematics, University of Birmingham, Birmingham, UK

**Keywords:** 35B40, 35Q92, 92D40

## Abstract

We study the long time behaviour of a non-local parabolic integro-differential equation modelling the evolutionary dynamics of a phenotypically-structured population in a changing environment. Such models can arise in variety of contexts from climate change to chemotherapy to the ageing body. The main novelty is that there are two locally optimal traits, each of which shifts at a possibly different linear velocity. We determine sufficient conditions to guarantee extinction or persistence of the population in terms of associated eigenvalue problems. When the population does not go extinct, we study the behaviour of long time solutions in the case of rare mutations: the long time solution concentrates as a sum of Dirac masses on a point set of "lagged optima" which are strictly behind the true shifting optima as the mutation rate goes to zero. If we further assume the shift velocities are different, we show the solution concentrates specifically on the positive lagged optimum with maximum lagged fitness. Our results imply that for populations undergoing competition in temporally changing environments, both the true optimal fitness and the required rate of adaptation for each of the diverging optimal traits determine the eventual dominance of one trait.

## Introduction

### Model and main questions

The non-local reaction-diffusion equation considered in this paper models an asexual population undergoing natural selection in a changing environment in the presence of multiple traits that give a locally optimal reproduction rate. The optima shift due to the changing environment, possibly with different velocities. Generally, we study models of the form

1 $$\begin{aligned} {\left\{ \begin{array}{ll} \partial _{t}n_{\varepsilon }(x,t)-\varepsilon ^2\partial _{xx}{n_{\varepsilon }(x,t)}=n_{\varepsilon }(x,t)\left( A(x,t)-\rho _\varepsilon (t)\right) ,& (x,t)\in {\mathbb {R}}\times {}[0,\infty ), \\ \rho _\varepsilon (t)=\int _{\mathbb {R}}n_{\varepsilon }(x,t)dx,& \\ n_{\varepsilon }(x,0)=n_{0}(x), & x\in \mathbb {R}, \end{array}\right. } \end{aligned}$$

where $n_\varepsilon (x,t)$ represents the concentration of individuals with trait $x\in \mathbb {R}$ present at time *t*. The function *A*(*x*, *t*) is the intrinsic growth rate of individuals with trait *x* at time *t*, where time dependence is due to the (slowly) changing environment. At a fixed time, the graph of *A*(*x*, *t*) is called the fitness landscape (Iglesias and Mirrahimi 2018; Lorenzi and Pouchol 2020; Iglesias and Mirrahimi 2021). The evolutionary concept of fitness is nuanced, but in the context of this work it can be regarded as synonymous with relative growth rate. The per capita growth rate, modified by competition, is given by ${A(x,t)}-\int _{\mathbb {R}}n_{{{\varepsilon }}}(y,t)dy$. The population dynamics are thus of Lotka-Volterra type where the competition is uniform across traits. Movement in trait-space is due to genetic alterations such as mutations. For simplicity, we model this using the diffusion term $\varepsilon ^2{\partial _{xx}{n}_\varepsilon }$, where $\varepsilon ^2$ is the mutation rate. This is a common assumption, but other works have also considered more general mutational kernels as in Carrillo et al. (2007) or Diekmann (2005).

Similar models have been studied under differing assumptions on the per capita growth term *A*(*x*, *t*) in many works (Lorenzi and Pouchol 2020; Iglesias and Mirrahimi 2021; Pouchol 2018; Magal and Webb 2000; Roques 2020). The case of multiple globally optimal traits is investigated in Lorenzi and Pouchol (2020) in a static environment. The case of a single optimal trait in a shifting and periodic environment is investigated in Iglesias and Mirrahimi (2021) which builds on the work (Iglesias and Mirrahimi 2018) that considers a periodic environment without shift. In the case of Iglesias and Mirrahimi (2021) the results pertain to the question of whether a species can adapt fast enough to a changing environment to survive. This question of persistence has also been investigated in Alfaro et al. (2017), which considers a spatially shifting environment and mutations in phenotype, and Berestycki and Fang (2018) in a purely spatial context. The authors of Iglesias and Mirrahimi (2021) find conditions on the rate of environmental shift and the maximum fitness to determine whether the population goes persists or not. They assume that the trait- and time-dependent per capita growth rate *A*(*x*, *t*) is given by a function ${a(x-\tilde{c}t,e(t))}$ where *e*(*t*) is a periodic function with period *T*, and the average fitness $\bar{a}(x)=\frac{1}{T}\int {}a(x,e(t))$ has just one global optimum. By using a growth function with this linearly shifting form, it is implicitly assumed that the changing environment affects locally optimal phenotypes equally. In other words, a given change in the environment displace all local optima equally. In reality, the trait and time-dependent per capita growth function may evolve in much more complicated ways.

In the present work, we take a form of the intrinsic growth rate that allows for two locally optimal traits each of which shifts linearly at possibly different rates. This leads to the following natural questions:

1. For which conditions on the shifting rate and intrinsic growth rates does the entire population go extinct?
2. Which traits dominate in the long time limit, if the population does not go extinct?

We are ultimately interested in the situation where *A*(*x*, *t*) has multiple time-dependent optima which shift at constant speeds with different directions, representing alternative evolutionary trajectories, i.e. representing alternative phenotypes which are adapted to the changing environment. To do this we study two distinct cases.

**Case 1:** For the case of multiple peaks shifting in the same direction at the same speed, the model (1) reduces to the following PDE:

2 $$\begin{aligned} {\left\{ \begin{array}{ll} \partial _{t}n_\varepsilon -\varepsilon ^2\partial _{xx}n_\varepsilon =n_\varepsilon \left( a(x-\tilde{c}t)-\rho _\varepsilon (t)\right) ,& (x,t)\in {\mathbb {R}}\times {}\mathbb {R}^{+}, \\ \rho _\varepsilon (t)=\int _{\mathbb {R}}n_\varepsilon (x,t)dx,\\ n(x,0)=n_{0}(x),& x\in \mathbb {R}. \end{array}\right. } \end{aligned}$$

Here $\tilde{c}>0$ can be interpreted as the rate at which the fitness of phenotypes responds to environment change, and it is necessary to choose the scaling $\tilde{c}=\varepsilon {}c$ since otherwise the population certainly goes extinct in the limit as $\varepsilon \rightarrow {{0}}$. To avoid technicalities, we assume that there are only finitely many maxima of *a*(*x*). We let $\{x_{1},x_{2},\dots ,x_{n}\}=\text {argmax}_{x\in \mathbb {R}} a(x)$, where $x_{i}$ are distinct points. We also assume there is a fixed ${d}>0$ such that $a(x)<-{d}$ for |*x*| sufficiently large. We let $a_{M}:=\max _{x\in \mathbb {R}}a(x)$.

**Case 2:** In the case where the optimal traits diverge, we will study the following PDE:

3 $$\begin{aligned} {\left\{ \begin{array}{ll} \partial _{t}n_\varepsilon -\varepsilon ^2\partial _{xx}{n}_\varepsilon =n_\varepsilon \left( a_{1}(x-\tilde{c}_{1}t)+a_{2}(x-\tilde{c}_{2}t)-d-\rho _\varepsilon (t)\right) , & (x,t)\in {\mathbb {R}}\times {}\mathbb {R}^{+}, \\ \rho _\varepsilon (t)=\int _{\mathbb {R}}n_\varepsilon (x,t)dx,\\ n_\varepsilon (x,0)=n_{0}(x). & \end{array}\right. } \end{aligned}$$

Here we assume $\tilde{c}_{1}<0<\tilde{c}_{2}$, and that *d* is a positive constant. We assume each $a_{i}$ is continuous, compactly supported in $[-R_{0},R_{0}]$, and has a unique maximum value $a_{i,M}$ at $x=x_{i}$. We again take the scaling $\tilde{c}_i=\varepsilon {}c_i$.

### Overview of results and structure of paper

The main results of the paper can be summarised as follows. For Case 1, Theorem 1 provides necessary and sufficient conditions on the eigenvalue of the linearised problem that determine if the population persists in the long time limit, and furthermore determines a point set on which it concentrates in the rare mutation limit when it does persist. We expect the set of concentration points can be further refined, and Theorem 2 shows that a particular weighted rescaling of the limiting solution will concentrate only on the shallowest peak, i.e. the $x_{i}\in \text {argmax}_{x\in \mathbb {R}}a(x)$ which minimises $|a''(x)|$. This suggests a peculiar result: the subpopulation which persists is the one which follows the shallowest moving optimum, even though that subpopulation may be itself concentrated at a trait where $|a''(x)|$ is large. This is not intuitive because it suggests the subpopulation benefits from the trait $x_{i}$ that none of the numbers of the limiting population have, since they are concentrated away from the moving optimum.

In Case 2, Theorem 3 again provides necessary and sufficient conditions on the population persistence, this time in terms of two related eigenvalue problems. When it does persists, we are able to reduce the problem to the single peak case and obtain an analogous result on convergence as in Theorem 1. The biological interpretation is more straightforward here: a subpopulation following a particular shifting optimal trait, even if not globally optimal, can persist provided the alternative optimal trait shifts fast enough.

The paper is structured as follows. In Section 2 we will present our main results in more detail, which answer the questions we have asked regarding persistence and extinction, and outline the strategy for proving them. In Section 3 we prove Theorems 1 to 3 by using Hamilton-Jacobi equations. In Section 4 we illustrate our results with numerical simulations (which provide some insight into the transient dynamics which are not captured by our theorems). In Section 5 we will discuss the biological interpretation of the results and suggest some future directions. For the sake of being self-contained, we collect relevant results from the literature in Section A. Moreover, this provides examples of the sort of results one can expect for this problem. We confine the more technical proofs to Appendix B.

### Biological relevance

Our results may be relevant for cancer biology, with regards to the phenomenon of decoy fitness peaks (Higa and DeGregori 2019). To summarise it, the hypothesis is that certain mutations (for instance in the NOTCH1 gene) may be non-cancerous but enable the mutants to survive better in an aged microenvironment allowing them to compete with pre-cancerous cells and thus suppress the development of tumours. Recent experimental evidence in support of this hypothesis can be found in Colom (2021). This phenomenon involves competition between two optimal phenotypes each of which is adapting to a time-dependent environment (in this case, due to ageing). Although our focus here is on the relevance of differing rates of adaptation, we suggest that this framework offers a useful starting point for modelling situations where asexual populations possessing multiple fit phenotypes are in competition in a temporally changing environment.

## Statement of results and strategy of proof

In this section, we set up the problem more thoroughly in each of the two cases and state our main theorems. We also detail our strategies for proving these results. In all cases, we analyse Hamilton-Jacobi equations associated with the problem. We derive relevant bounds on the solution to the PDE by using the notion of generalised super- and sub-solutions as provided in Lam and Lou (2022), and the results on principal Floquet bundles from Húska and Polacik (2008). These results are reviewed in, respectively, Appendix A.2 and Appendix A.3.

### Case 1: Two peaks shifting in with the same velocity

After taking the appropriate scaling of the drift term, system (2) may be written

4 $$\begin{aligned} {\left\{ \begin{array}{ll} \partial _{t}n_\varepsilon -\varepsilon ^{2}\partial _{xx}n_\varepsilon =n_\varepsilon \left( a(x-c\varepsilon {t})-\rho _\varepsilon (t)\right) , & (x,t)\in {\mathbb {R}}\times {}\mathbb {R}^{+}, \\ \rho _\varepsilon (t)=\int _{\mathbb {R}}n_\varepsilon (x,t)dx,\\ n_\varepsilon (x,0)=n_{0}(x). \end{array}\right. } \end{aligned}$$

We make the following assumptions:

1. There exist $R_{0},{d}>0$ such that $a(x)<-{d}$ provided $|x|>R_{0}$
2. $$a(x)\in C^{2}(\mathbb {R})$$, $$\max _{x\in {}\mathbb {R}}a(x)=a_M<\infty $$, $${\min {}_{x\in \mathbb {R}}a(x)\ge {}a_m>-\infty }$$
3. $0\le {}n_{0}\le {}e^{C_{1}-C_2|x|}$ for some positive constants $C_{1}$ and $C_{2}$, and $n_{0}\in {}C(\mathbb {R}).$
4. There are a finite number of global maxima of *a*.

To state the next, and final, assumption succinctly, we must introduce some notation. We recall $M=\{x_{1},x_{2},\dots ,x_{n}\}=\text {argmax}_{x\in \mathbb {R}}a(x)$, and that $a_{M}$ is the maximum of *a*(*x*). We assume the optima are ordered $x_{i}<x_{i+1}$ for each $i=1,\ldots ,n-1$. Our final assumption is then

(A5) For $i=2,\ldots ,n$ there are only two solutions to $a(x)=a_{M}-\frac{c^2}{4}$ in the interval $(x_{i-1},x_{i})$, letting $\bar{x}_{i}$ be the greatest of these, and that there is just one solution $\bar{x}_{1}$ in the interval $(-\infty ,x_{1})$.

In preparation for the statement of our first result, we first consider some transformed problems and the associated eigenvalue problems. Firstly, we take $N_\varepsilon (x,t)=n_\varepsilon (x+\varepsilon {c}t,t)$ so that $N_\varepsilon (x,t)$ solves:

5 $$\begin{aligned} {\left\{ \begin{array}{ll} \partial _{t}N_\varepsilon -\varepsilon ^{2}\partial _{xx}N_\varepsilon -\varepsilon {c}\partial _{x}N_\varepsilon =N_\varepsilon \left( a(x)-{\rho _\varepsilon (t)}\right) , & (x,t)\in {\mathbb {R}}\times \mathbb {R}^{+}, \\ N_\varepsilon (x,0)=n_{0}(x). \end{array}\right. } \end{aligned}$$

Next one can linearlise this by working with the equation for $m_\varepsilon (x,t)=N_\varepsilon {}(x,t)e^{\int _{0}^{t}\rho _{\varepsilon }(s)ds}$.

6 $$\begin{aligned} {\left\{ \begin{array}{ll} \partial _{t}m_\varepsilon -c\varepsilon \partial _{x}m_\varepsilon -\varepsilon ^2\partial _{xx}m_\varepsilon =a(x)m_\varepsilon , & {x}\in {\mathbb {R}}, \\ m_\varepsilon (x,0)=n_{0}(x).\\ \end{array}\right. } \end{aligned}$$

Finally, one can remove the drift term by applying a Liouville transform $M_\varepsilon (x,t)=m_\varepsilon (x,t)e^\frac{cx}{2\varepsilon }$:

7 $$\begin{aligned} {\left\{ \begin{array}{ll} \partial _{t}M_\varepsilon -\varepsilon ^2\partial _{xx}M_\varepsilon =M_\varepsilon \left( a(x)-\frac{{c}^2}{4}\right) , & (x,t)\in {\mathbb {R}}\times \mathbb {R}^{+}, \\ M_\varepsilon (x,0)=n_{0}(x)e^{\frac{{c}x}{2\varepsilon }}.\\ \end{array}\right. } \end{aligned}$$

Associated to (6) and (7) (respecitvely) are the following stationary eigenvalue problems

8 $$\begin{aligned} {\left\{ \begin{array}{ll} -\varepsilon ^2\partial _{xx}{{p}_\varepsilon }-\varepsilon {}c\partial _{x}{p}_\varepsilon -a(x){{p}_\varepsilon }={\lambda }_{\varepsilon }{{p}_\varepsilon }, & x\in {\mathbb {R}}, \\ {p_\varepsilon }>0, & \end{array}\right. } \end{aligned}$$

and

9 $$\begin{aligned} {\left\{ \begin{array}{ll} -\varepsilon ^2\partial _{xx}{\hat{p}_\varepsilon }-\left( a(x)-\frac{c^2}{4}\right) {\hat{p}_\varepsilon }={\lambda }_{\varepsilon }{\hat{p}_\varepsilon }, & x\in {\mathbb {R}}, \\ {\hat{p}_\varepsilon }>0. & \end{array}\right. } \end{aligned}$$

**Fig. 1:**
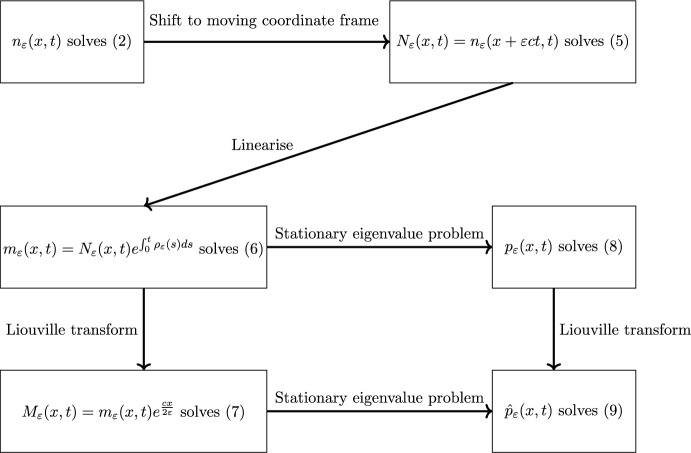
A map of the various transformed problems, eigenvalue problems, and the relations between them

See Figure 1 for a map of the relationships between the transformations and eigenvalue problems.

Our main theorem says that the solution of (5) at long times is approximately given by a multiple of eigenvector which solves (8), and does not go extinct if the eigenvalue is negative. The eigenvalue itself is shown to converge to $-\left( a_{M}-\frac{c^2}{4}\right)$ so for sufficiently small $\varepsilon$ we can determine its sign.

#### Theorem 1

Under the assumptions (A1) to (A5):

Suppose that ${p}_\varepsilon (x)$ is the unique $L_1$ normalised solution to (8), and for each $i=1,\ldots ,n$ that ${\alpha _i}$ is a non-negative constant, such that $\sum _{i=1}^{n}{\alpha _i}=1$. Then we haveIf $\lambda _{\varepsilon }<0$, then we also haveandIf $\lambda _\varepsilon \ge {}0$, then .

Here $\delta _z$ is the Dirac delta measure centred at *z*.

This theorem generalises some of the results from Lorenzi and Pouchol (2020) and Iglesias and Mirrahimi (2021); specifically it characterises the long time behaviour of solutions in the novel case of a linearly shifting growth rate which has multiple global optima. We refer the point set $\{\bar{x}_1,\ldots ,\bar{x}_n\}$ as the set of lagged optima since each $\bar{x}_i$ is a candidate location for the solution $p_\varepsilon$ to concentrate.

To prove Theorem 1, we use the theory developed in Húska and Polacik (2008), which we review in Appendix A.3, to determine the long time behaviour of solutions to (6) in terms of the solution to the associated eigenvalue problem (8). The solutions of the eigenvalue problems are analysed using the Hamilton-Jacobi approach.

Although this theorem gives some information about the limiting solution we expect it can be made stronger by further restricting the set of points where the solution concentrates. This will be shown via numerical simulations. We note that in Lorenzi and Pouchol (2020) the set of limit points is refined further, according to Proposition 3 in Lorenzi and Pouchol (2020) (restated as Lemma 16 in Section A).

While unable to fully classify the limit measure we are able to show the convergence of the transformed and rescaled eigenvalue defined as

$$\begin{aligned} {P_\varepsilon }=\frac{{p}_\varepsilon e^{\frac{cx}{2\varepsilon }}}{\Vert {p}_\varepsilon e^{\frac{cx}{2\varepsilon }}\Vert _{L_{1}(\mathbb {R})}}, \end{aligned}$$

which we will show is the $L_1$ normalised solution to (9) in Section 3. In particular, we show that $\Vert {}{p}_\varepsilon {}e^{\frac{cx}{2\varepsilon }}\Vert _{L_{1}(\mathbb {R})}$ is finite. Then by borrowing a result from semi-classical analysis (as is done in Lorenzi and Pouchol (2020)) we prove our next theorem.

#### Theorem 2

Let $S(x)=|a''(x)|$ for $x\in {}\{x_{1},\ldots ,x_{n}\}=\text {argmax}_{x\in \mathbb {R}}a(x)$. Where $\text {argmin}_{x_{j}}S(x_{j})=\{x_{i_{1}},x_{i_{2}},\ldots ,x_{i_{k}}\}$ we have:

Up to extraction of subsequences,where $\alpha _{j}>0$ for each *j* and $\sum _{j=1}^{k}{\alpha _{j}}=1$.

The main difficulty in proving Theorem 2 lies in the fact that we work on an unbounded domain, whereas Proposition 3 from Lorenzi and Pouchol (2020) is obtained for problems in a bounded domain (or on a compact Riemannian manifold in Holcman and Kupka (2006)). To overcome this, we will use the results in Húska and Polacik (2008) to construct the solution to (9) as the limit of solutions to the following Dirichlet problems on the balls $B_R=[-R,R]$.

10 $$\begin{aligned} {\left\{ \begin{array}{ll} -\varepsilon ^2\partial _{x{x}}\hat{p}_{R,\varepsilon }-\left( a(x)-\frac{c^2}{4}\right) \hat{p}_{R,\varepsilon }=\lambda _{R,\varepsilon }\hat{p}_{R,\varepsilon }, & (x,t)\in {}B_{R}\times {\mathbb {R}^{+}}, \\ \hat{p}_{R,\varepsilon }>0, & (x,t)\in {}B_{R}\times {\mathbb {R}^{+}},\\ \hat{p}_{R,\varepsilon }=0, & (x,t)\in {}\partial {}B_{R}\times {\mathbb {R}^{+}}. \end{array}\right. } \end{aligned}$$

Such a construction is also used in Iglesias and Mirrahimi (2021), and in our proof of Theorem 1. Fortunately, this procedure also enables us to approximate the eigenfunction-eigenvalue pair $({\hat{p}_\varepsilon },\lambda _\varepsilon )$ in terms of $({\hat{p}_{R,\varepsilon }},\lambda _{R,\varepsilon })$. We can apply the same semi-classical analysis results as used in Lorenzi and Pouchol (2020), which require us to work in a bounded domain, to these approximate problems, allowing us to refine the set of concentration points of ${\hat{p}_\varepsilon }$ as $\varepsilon \rightarrow {0}$.

The procedure of estimating the problem on the whole real line can similarly be used to estimate $\lambda _\varepsilon$ and obtain its limiting value as $\varepsilon \rightarrow {0}$ which will be used to determine the limiting set. We show later that the convergence of $\lambda _{R,\varepsilon }$ as $\varepsilon \rightarrow {0}$ is uniform in *R*, which allows us to adapt the methods used in Lorenzi and Pouchol (2020) to obtain the required concentration result.

### Case 2: Two peaks shifting at different speeds

In this case, we consider (1) and set $a(x,t)=a_{1}(x-\varepsilon {c}_{1}t)+a_{2}(x-\varepsilon {c}_{2}t)-{d}$. We arrive at the equation

11 $$\begin{aligned} {\left\{ \begin{array}{ll} \partial _{t}n_\varepsilon -\varepsilon ^2{\partial _{xx}}{n_\varepsilon }=n_\varepsilon \left( a_{1}(x-\varepsilon {c}_{1}t)+a_{2}(x-\varepsilon {c}_{2}t)-{d}-\rho _\varepsilon (t)\right) , & (x,t)\in {\mathbb {R}}\times {}\mathbb {R}^{+}, \\ {\rho _\varepsilon (t)=\int _{\mathbb {R}}n_\varepsilon (x,t)dx},\\ n_\varepsilon (x,0)=n_{0}(x). \end{array}\right. } \end{aligned}$$

Here we assume the following:

1. The functions $a_{1}$ and $a_{2}$ are in $C^{{2}}_{c}([-R_{0},R_{0}])$, $\max _{\mathbb {R}}a_{i}(x)=a_{i,M}<\infty$, and $\min _{x\in \mathbb {R}}a_i(x)>a_m>-\infty$.
2. The shifting rates satisfy $c_{1}<0<c_{2}$.
3. $0\le {}n_{0}\le {}e^{C_{1}-C_{2}|x|}$ for some positive constants $C_{1}$ and $C_{2}$, and ${n_0\in {}C(\mathbb {R})}$
4. There are finitely many global maxima for each $a_{i}(x)$.

We assume the global maxima of $a_1(x)$ are ordered $x^{1}_j>x^{1}_{j+1}$, and the global maxima of $a_2(x)$ are ordered $x^{2}_j<x^{2}_{j+1}$, for $j=1,\ldots ,n_j-1$.

(B5) For $j=2,\ldots ,n_1$ there are only two solutions to $a_1(x)=a_{1,M}-\frac{c_1^2}{4}$ in the interval $(x^{1}_{j},x^{1}_{j-1})$ letting $\bar{x}^1_j$ be the least of these, and that there is just one solution $\bar{x}^i_1$ in $(x_1^i,\infty )$. Analogously, we assume there are only two solutions to $a_2(x)=a_{2,M}-\frac{c_2^2}{4}$ in the interval $(x^{2}_{j-1},x^2_{j})$, letting $\bar{x}^2_j$ be the greatest of these, and that there is only one solution $\bar{x}_1^2$ in $(-\infty ,x_1^2)$

If one of $a_i$ were identically zero, (B4) would be equivalent to (A4). Note that because $c_1<0$ the assumption (B5) on $a_1$ is equivalent to (A5) on *a* if we transform $x\rightarrow {-x}$.

This model represents mutation and competition in the presence of two alternative shifting optima in the fitness landscape. In other words, at a given time *t*, there are two locally optimum traits, $x_{1}+\varepsilon {c_1}t$ and $x_{2}+\varepsilon {c_2}t$. In light of the previous results, we expect to obtain conditions on $a_{i,M}$ and $c_{i}$ that determine whether the population goes extinct or not, and where the population concentrates if it does not.

To state our main result for this case, we need the following transformed problems. In what follows, we sometimes suppress the $\varepsilon$ subscripts for ease of notation. We let $N_{i}(x,t)=n(x+\varepsilon {}c_{i}t,t)$, $C=c_{2}-c_{1}$ and $m_{i}=N_{i}(x,t)e^{\int _{0}^{t}\rho _{\varepsilon }(s)ds}$. Then $m_{i}$ solves:

$$\begin{aligned} {\left\{ \begin{array}{ll} \partial _{t}m_{i}-\varepsilon ^2\partial _{xx}{m}_{i}-\varepsilon {}c_{i}\partial _{x}m_{i}=m_{i}\left( a_{i}(x)+a_{i'}(x+(-1)^{i}\varepsilon {C}t)-{d}\right) , & ~~(x,t)\in {\mathbb {R}}\times {}\mathbb {R}^{+}, \\ m_{i}(x,0)=n_{0}(x). \end{array}\right. } \end{aligned}$$

where $i'$ is 1 if $i=2$ and 2 if $i=1$. Repeating similar transformations as before, by letting ${M}_{i}=m_{i}e^{\frac{c_{i}x}{2\varepsilon }}$, we find that ${M}_{i}$ solves

$$\begin{aligned} {\left\{ \begin{array}{ll} \partial _{t}{M}_{i}-\varepsilon ^2\partial _{xx}{{M}_{i}}={M}_{i}\left( a_{i}(x)+a_{i'}(x+(-1)^{i}\varepsilon {C}t)-\frac{c_{i}^2}{4}-{d}\right) , & (x,t)\in {\mathbb {R}}\times {}\mathbb {R}^{+}, \\ {M}_{i}(x,0)=n_{0}(x)e^{\frac{c_{i}x}{2\varepsilon }}. \end{array}\right. } \end{aligned}$$

We also need the following eigenvalue problems. For $i\in {1,2}$,

12 $$\begin{aligned} &  {\left\{ \begin{array}{ll} -\varepsilon ^2\partial _{xx}{p}_{i,\varepsilon }-\varepsilon _{}c_{i}\partial _{x}{p}_{i,\varepsilon }={p}_{i,,\varepsilon }\left( \lambda _{i,\varepsilon }+a_{i}(x)-{d}\right) , & x\in {\mathbb {R}}, \\ p_{i,\varepsilon }>0 & x\in {\mathbb {R}}, \end{array}\right. } \end{aligned}$$

13 $$\begin{aligned} &  {\left\{ \begin{array}{ll} -\varepsilon ^2\partial _{xx}{{\hat{p}_{i,\varepsilon }}}={\hat{p}_{i,\varepsilon }}\left( \lambda _{i,\varepsilon }+a_{i}(x)-\frac{c_{i}^2}{4}-{d}\right) , & x\in {\mathbb {R}}, \\ {\hat{p}_{i,\varepsilon }}>0 & x\in {\mathbb {R}}.\\ \end{array}\right. } \end{aligned}$$

We note that, once we fix a normalisation of $p_{i,\varepsilon }$, we have that ${{\hat{p}_{i,\varepsilon }}}=w_\varepsilon {}p_{i,\varepsilon }e^{\frac{c_ix}{2\varepsilon }}$ for some positive constant $w_\varepsilon$.

We proceed similarly to Iglesias and Mirrahimi (2018), and obtain estimates on local growth rate near each of the dominant traits. Our main result determines conditions for the survival of the population in the long time. In particular, the long time behaviour is determined by the minimal eigenvalue. As such, we let $\underline{i}\in \{1,2\}$ be such that $\lambda _{\underline{i},\varepsilon }=\min \{\lambda _{1,\varepsilon },\lambda _{2,\varepsilon }\}$, and $\overline{i}\in \{1,2\}$ be such that $\lambda _{\overline{i},\varepsilon }=\max \{\lambda _{1,\varepsilon },\lambda _{2,\varepsilon }\}.$

#### Theorem 3

Under the assumptions (B1) to (B5), we have for $i\in \{1,2\}$where the constants $\alpha _{j}^i$ are non-negative with $\sum _{j=1}^{n_i}\alpha _j^i=1.$

If $\lambda _{\underline{i},\varepsilon }<0$ and $\lambda _{1,\varepsilon }\ne {}\lambda _{2,\varepsilon }$ we haveandIf instead $\lambda _{\underline{i},\varepsilon }\ge {0}$ then  .

#### Remark 1

In the special case where $a_{\underline{i},M}(x)$ has a unique global maximum $x^{\underline{i}}_1$, the solution must concentrate at exactly $\bar{x}^{\underline{i}}_1$. Also Theorem 2 applies to the eigenfunctions ${\hat{p}_{i,\varepsilon }}$, and could be a useful step towards further refining the concentration points.

To prove Theorem 3 we use a comparison theorem to construct a supersolution that vanishes on the support of $a_{\overline{i}}(x-\varepsilon {}c_2t)$. The long time behaviour is consequently the same as in the one peak case where $a(x)=a_{\underline{i}}(x-\varepsilon {}c_{\underline{i}}t)-{d}$, and so we may apply Theorem 1. To end this section, we will offer some heuristics in support of Theorem 3. Suppose that, as might be expected from Iglesias and Mirrahimi (2018), we have obtained the local growth rates in a ball of radius *R*:When extinction does not occur, we expect that  for some positive constant, and, of course that $0<\Vert {}n\Vert _{L^{\infty }(\mathbb {R})}<\infty$. These facts together mean the only possible value of $\rho _\infty$ is $\text {max}(-\lambda _{1,\varepsilon },-\lambda _{2,\varepsilon })$, since any other value would mean either extinction or unbounded growth.

## Proofs of main results

**Fig. 2:**
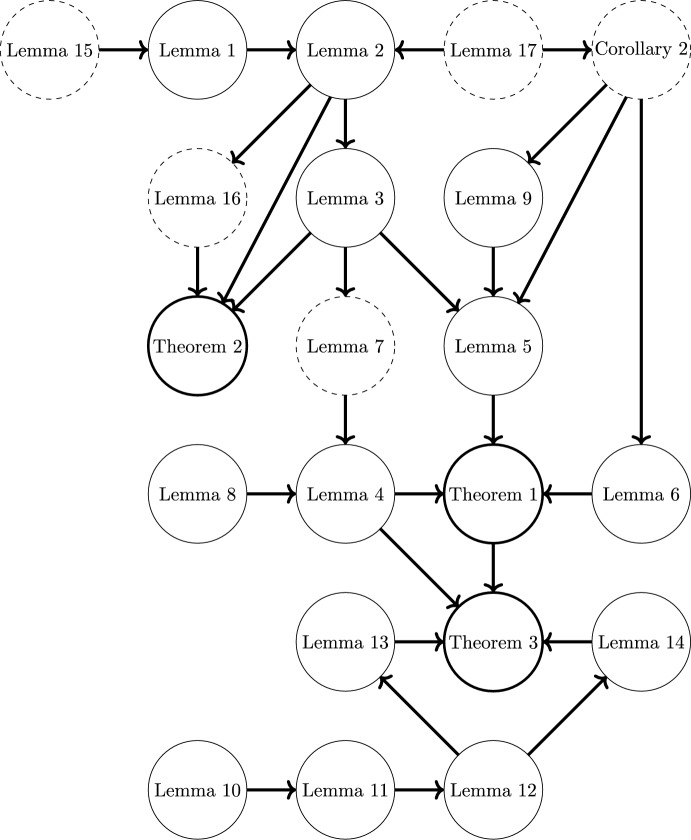
Map showing the dependencies of each lemma and theorem. Dashed circles correspond results which are proved in the Appendix, thin solid circles correspond to lemmas proved in this section, and thick solid lines correspond to the main theorems

In this section we prove the main results and several auxiliary lemmas. See Figure 2 for a guide to dependences of the Lemmas and theorems.

### Proof of Theorems 1 and 2

Theorem 1 is a direct consequence of Lemmas 4 to 6 below. A solution $p_\varepsilon$ to (8) is obtained as consequence of Lemma 2..

#### Lemma 1

Let $\lambda _{R,\varepsilon }$ be the eigenvalue from (10). Then $\lambda _{R,\varepsilon }$ is monotonically decreasing in *R*, and  where this convergence is uniform in *R*.

#### Lemma 2

The solutions ${\hat{p}_{R,\varepsilon }}$ to (10) converge locally uniformly in $\mathbb {R}$ to a solution ${\hat{p}_\varepsilon }$ to (9), and 

If $\hat{p}_\varepsilon$ is a solution to (9), then $p_\varepsilon =e^{-\frac{cx}{2\varepsilon }}\hat{p}_\varepsilon$ is a solution to (8). The next lemma shows that both eigenfunctions can be normalised.

#### Lemma 3

Let $p_\varepsilon ^{\infty }$ be the solution to (8) such that $\Vert {}p_\varepsilon ^\infty \Vert _{L^\infty (\mathbb {R})}=1$. We have the following bounds:

$$\begin{aligned} e^{-\frac{\underline{\kappa }|x-x_\varepsilon |}{\varepsilon }}\le p_{\varepsilon }^{\infty }(x)\le \min \left\{ 1,e^{\frac{-\overline{\kappa }(|x|-R_0)}{\varepsilon }}\right\} ,~~x\in \mathbb {R}, \end{aligned}$$

where $\underline{\kappa }=\frac{c}{2}{+2\sqrt{\left| a_m+\lambda _{\varepsilon }-\frac{c^2}{4}\right| }}$, and $\overline{\kappa }={\frac{\sqrt{a_M}+c}{2}}$, and $x_\varepsilon$ is a point where $p_\varepsilon (x_\varepsilon )=1$.

From here we fix $p_\varepsilon$ as the solution to (8) such that $\Vert {p_\varepsilon }\Vert _{L^{1}(\mathbb {R})}=1$.

#### Lemma 4

Assume (A1),(A2),(A4),(A5), thenwhere the $\alpha _{i}$ are nonegative and $\sum _{i=1}^{n}\alpha _i=1$.

#### Lemma 5

Under the assumptions $a_{M}-\frac{c^2}{4}>0$, $\varepsilon$ is small enough, and (A1)-(A5) the normalized population will converge to $p_\varepsilon$ as $t\rightarrow \infty$, i.e.

#### Lemma 6

Under the assumptions $a_{M}-\frac{c^2}{4}>0$, $\varepsilon$ is small enough, and (A1)-(A5) the total population $\rho _\varepsilon (t)$ will convergence to a finite, positive value as $t\rightarrow {\infty }$. That is,

Theorem 2 will be proved using Lemma 16 (in Appendix A.3) and Lemma 2.

We begin with the proof of Lemma 1 since we will require the limiting value of $\lambda _\varepsilon$ as $\varepsilon \rightarrow {0}$.

#### Proof of Lemma 1

It is clear that $\lambda _{R,\varepsilon }\ge {-\left( a_{M}-\frac{c^2}{4}\right) }$ using the Rayleigh-Quotient:

$$\begin{aligned} \lambda _{R,\varepsilon }=\inf _{\phi \in {H}^{1}_{0}(B_{R})\backslash {0}}\frac{\varepsilon ^2\int _{B_{R}}|\partial _{x}\phi |^2-\int _{B_{R}}\left( a(x)-\frac{c^2}{4}\right) \phi ^2}{\int _{B_{R}}\phi ^2}. \end{aligned}$$

The same formula shows that $\lambda _{R,\varepsilon }$ is monotonically decreasing in *R*, since $H_0^1(B_R)\subset {}H_0^1(B_{R'})$ for $R'>R$. We need to pick a sequence of $\phi _\varepsilon \in {H^{1}_{0}}(B_{R})\backslash \{0\}$ such thatWe cannot use the same sequence as in Lorenzi and Pouchol (2020) since their functions do not vanish on the boundary, and are hence not in $H_{0}^{1}(B_{R}).$ Instead we do the following. Let $\chi$ be a smooth cut off function such that $\chi (x)=0$ for $|x|>1$, and $\chi (x)=1$ for $x\in (-\frac{1}{2},\frac{1}{2})$. Then, similarly to Lorenzi and Pouchol (2020), take $G=D_{1}\chi ^2{}e^{-|x|^2}$ where $D_{1}$ is a normalising constant.

Define the sequence $\phi _\varepsilon ^2=\frac{1}{\varepsilon ^\frac{1}{2}}G\left( \frac{x-x_{i}}{\varepsilon ^{\frac{1}{2}}}\right)$ for $x_{i}\in {}\text {argmax}_{x\in \mathbb {R}}\left( a(x)-\frac{c^2}{4}\right)$. We have (due to the normalisation) that . The choice of $x_{i}$ is arbitrary, so it is sufficient to find a sequence of functions which concentrate on just one of the global optima. Thus, we only need to check that . We compute

$$\begin{aligned} |\partial _{y}\phi _{\varepsilon }(y)|^2=\varepsilon ^{-\frac{3}{2}}\left| (G^{\frac{1}{2}})'\left( \frac{y-x_{i}}{\varepsilon ^{\frac{1}{2}}}\right) \right| ^2, \end{aligned}$$

which is sufficient since

$$\begin{aligned} \int _{B_{R}}\left| (G^{\frac{1}{2}})'\left( \frac{y-x_{i}}{\varepsilon ^{\frac{1}{2}}}\right) \right| ^2dy&\le {} \int _{\mathbb {R}}\left| (G^{\frac{1}{2}})'\left( \frac{y-x_{i}}{\varepsilon ^{\frac{1}{2}}}\right) \right| ^2dy\\&\le {}\varepsilon ^{\frac{1}{2}}\int _{\mathbb {R}}|(G^{\frac{1}{2}})'(y)|^2dy \end{aligned}$$

and $\int _{\mathbb {R}}|(G^{\frac{1}{2}})'(y)|^2dy$ is a fixed constant.

Hence we get that . We note that we can pick the same $\phi _\varepsilon ^2$ independently of *R* (supposing *R* is large enough) and so this convergence is independent of *R*. $\square$

We next prove Lemma 2 which allows us to use $p_{R,\varepsilon }$ to prove the existence of, and estimate, $p_\varepsilon$

#### Proof of Lemma 2

Let $\tilde{p}_{R,{K},\varepsilon }=e^{({K}-\lambda _{R,\varepsilon })t}{\hat{p}_{R,\varepsilon }}$ which solves:

14 $$\begin{aligned} {\left\{ \begin{array}{ll} \partial _{t}\tilde{p}_{R,\varepsilon }-\varepsilon ^2\partial _{xx}\tilde{p}_{R,\varepsilon }-\left( K+a(x)-\frac{c^2}{4}\right) \tilde{p}_{R,\varepsilon }=0 & \text { in } B_{R}, \\ \tilde{p}>0, & \\ \tilde{p}=0 & \text { on } \partial {}B_{R}, \end{array}\right. } \end{aligned}$$

and impose that $\Vert {}\tilde{p}_{R,K,\varepsilon }(x,0)\Vert _{L^\infty ({\mathbb {R}})}=\Vert {}\hat{p}_{R,\varepsilon }(x,0)\Vert _{L^\infty ({\mathbb {R}})}=1$.

This is a particular case of the sequence of Dirichlet problems considered in Húska and Polacik (2008). We only need to check the exponential separation property, which follows straightforwardly from the formula $\tilde{p}_{R,{K}{,}\varepsilon }=e^{{(K}-\lambda _{R,\varepsilon }{)}t}{\hat{p}_{R,\varepsilon }}$, given we pick $K>\lambda _{R,\varepsilon }$. Hence, by Lemma 17, a subsequence $\tilde{p}_{R_{n},{K},\varepsilon }$ converges locally uniformly in $\mathbb {R}\times \mathbb {R}$ to $\tilde{p}_{{K},\varepsilon }$ which solves

15 $$\begin{aligned} {\left\{ \begin{array}{ll} \partial _{t}\tilde{p}_{{K},\varepsilon }-\varepsilon ^2\partial _{xx}\tilde{p}_{{K},\varepsilon }-\left( K+a(x)-\frac{c^2}{4}\right) \tilde{p}_{{K},\varepsilon }=0 & \text { in } \mathbb {R}\times {}\mathbb {R} \\ {\tilde{p}}_{{K,\varepsilon }}>0, & {} \end{array}\right. } \end{aligned}$$

From Lemma 1 we have that $\lambda _{R,\varepsilon }$ is monotonic *R* and bounded. Consequently $\lambda _{R,\varepsilon }$ converges as $R\rightarrow {\infty }$ to a limiting value $\lambda _\varepsilon$, and that . Thus:We let $\hat{p}_\varepsilon (x,t)=e^{(-K+\lambda _\varepsilon {})t}\tilde{p}_{K,\varepsilon }$ which satisfies

16 $$\begin{aligned} {\left\{ \begin{array}{ll} \partial _{t}\hat{p}_\varepsilon -\varepsilon ^2\partial _{xx}\hat{p}_\varepsilon -\left( a(x)-\frac{c^2}{4}\right) \hat{p}_\varepsilon ={\lambda }_{\varepsilon }\hat{p}_\varepsilon , & x\in {\mathbb {R}}, \\ {\hat{p}_\varepsilon }>0. & {} \end{array}\right. } \end{aligned}$$

which is the time dependent version of (9). But $\hat{p}_\varepsilon (x,t)=\hat{p}_\varepsilon (x)$ is independent of time since it is the limit of time-independent functions, and consequently $\hat{p}_\varepsilon (x)$ is a solution to (9) $\square$

We can now prove Lemma 3.

#### Proof of Lemma 3

We let $\mathcal {L}(u):=-\varepsilon ^2\partial _{xx}u-c\varepsilon {}\partial _{x}u-(a(x)+\lambda _\varepsilon )u$. The first inequality is a consequence the comparison theorem in Section A that applies once we show $e^{\frac{-\overline{\kappa }(|x|-R_0)}{\varepsilon }}$ is a generalised supersolution of (8) on $\mathbb {R}/B_{R_0}$. Since, $p_{\varepsilon }^\infty (\pm {}R_0)\le {}1$ the boundary condition is satisfied and we only need to check that $\mathcal {L}\left( e^{\frac{-\overline{\kappa }(|x|-R_0)}{\varepsilon }}\right) \ge {0}$ for $|x|>R_0$. This reduces to $-\overline{\kappa }^2{+}c\overline{\kappa }-(-{d}+\lambda _\varepsilon )\ge {0}$. Since  we see, for sufficiently small $\varepsilon$, that it is sufficient if $\overline{\kappa }\in (\frac{c}{2}-\sqrt{a_M},\frac{c}{2}+\sqrt{a_M})$, which is satisfied by $\overline{\kappa }=\frac{\sqrt{a_M}+c}{2}$.

The lower bound is similar, except the sufficient condition is $-\underline{\kappa }^2+c\underline{\kappa }-({a_m}+\lambda _\varepsilon )\le {}0$, which follows from (A2). One checks that for $\underline{\kappa }=\frac{c}{2}{+2\sqrt{\left| a_m+\lambda _{\varepsilon }-\frac{c^2}{4}\right| }}$ the inequality is satisfied. $\square$

#### Remark 2

Since $\overline{\kappa }>\frac{c}{2}$ this implies that $p_\varepsilon (x)e^{\frac{cx}{2\varepsilon }}$ is integrable.

Using this result, we can prove Theorem 2.

#### Proof of Theorem 2

The expression

$$\begin{aligned} \hat{p}_\varepsilon =\frac{{p}_\varepsilon e^{\frac{cx}{2\varepsilon }}}{\Vert {p}_\varepsilon e^{\frac{cx}{2\varepsilon }}\Vert _{L_{1}(\mathbb {R})}}, \end{aligned}$$

is merely the $L_{1}$ normalised solution ${\hat{p}_\varepsilon }$ to (9), and the normalisation is possible because of Lemma 3. According to Lemma 16, the functions ${\hat{p}_{R,\varepsilon }}$, similarly normalized in $L^{1}$, satisfy:where $\{x_{i_1},x_{i_2},\ldots ,x_{i_k}\}=\text {argmax}_{z\in \mathbb {R}}a(x)\cap \text {argmin}_{x\in \text {argmax}_{x\in \mathbb {R}}a(x)}|a''(z)|$. But ${\hat{p}_{R,\varepsilon }}$ approaches $\hat{p}_{\varepsilon }$ locally uniformly according to Lemma 2. Hence if ${\hat{p}_{R,\varepsilon }}\rightarrow {0}$ as $\varepsilon \rightarrow {0}$ in some bounded open set *U*, then the same is true for $\hat{p}_{\varepsilon }$. This completes the proof. $\square$

Next, we proceed with the proof of Theorem 1, but require some more set up to do so. Our approach here is similar to that in Iglesias and Mirrahimi (2021) except that we are working directly with viscosity solutions to the (time-independent) eigenvalue problem rather than with the original PDE. We will first apply the WKB ansatz. Letting $\psi _\varepsilon$ satisfy,

$$\begin{aligned} {p}_{\varepsilon }=e^{\frac{\psi _{\varepsilon }}{\varepsilon }}. \end{aligned}$$

We find that $\psi _{\varepsilon }$ solves

$$ -\varepsilon \partial _{xx}\psi _\varepsilon -\left| \partial _{x}\psi _{\varepsilon }+\frac{c}{2}\right| ^2-a(x)+\frac{c^2}{4}-\lambda _{\varepsilon }=0.$$

We will show, analogously to Theorem 1.1 in Iglesias and Mirrahimi (2021), the following lemma

#### Lemma 7

Assuming (A1), (A2), (A4) and (A5) the function $\psi _\varepsilon$ converges (up to extraction of subsequences) locally uniformly to a viscosity solution $\psi$ of

17 $$\begin{aligned} {\left\{ \begin{array}{ll} -\left| \partial _{x}\psi +\frac{c}{2}\right| ^2+a_M-a(x)=0,\\ \max _{x\in \mathbb {R}}\psi =0.\\ \end{array}\right. } \end{aligned}$$

We defer the proof to the appendix.

A corollary of Lemma 7 is that ${p}_\varepsilon$ concentrates on the set of points such that $\psi =0$.

#### Corollary 1

Assume there are only finitely many points solving $\psi (x)=0$. Let these be $x_{1}',x_{2}',\ldots ,x_{k}'$. For the function ${p}_\varepsilon$ we have, after an extraction of subsequenceswhere ${\alpha }_{i}\ge {}0$ for each *i* and $\sum _{i}{\alpha }_{i}=1$.

Note that we can also prove that ${p}_\varepsilon$ concentrates on the finitely many points by following Proposition 1 in Lorenzi and Pouchol (2020). Unfortunately, this method includes more concentration points than expected, since it does not exclude the smallest value $y_{i}$ which satisfies both $y_{i}>x_{i}$ and $a(y_{i})=a_{M}-\frac{c^2}{4}$. We can get a more refined result using the Hamilton-Jacobi equation method. Thus we consider the equation solved by $u=\psi (x)+\frac{cx}{2}$, which is:

18 $$\begin{aligned} {\left\{ \begin{array}{ll} -\left| \partial _{x}u\right| ^2+a_M-a(x)=0,\\ \max _{x\in \mathbb {R}}u-\frac{cx}{2}=0.\\ \end{array}\right. } \end{aligned}$$

We now find the possible set of points for which $\psi =0$. We do not aim to show the uniqueness of solutions, only refine the set of concentration points. It is straightforward to verify that viscosity solutions of (18) are also viscosity solutions of

19 $$\begin{aligned} {\left\{ \begin{array}{ll} -\left| \partial _{x}u\right| +\sqrt{a_M-a(x)}=0,\\ \max _{x\in \mathbb {R}}u-\frac{cx}{2}=0.\\ \end{array}\right. } \end{aligned}$$

We recall a well-known result for viscosity solutions of Hamilton-Jacobi equations which is proved in Crandall and Lions (1984). Let $\Omega \subset {\mathbb {R}}$ be a bounded domain and $n(x)\in {C}\left( \bar{\Omega }\right)$ and $n>0$. Consider the equation:

20 $$\begin{aligned} \left\{ \begin{array}{l} |\partial _{x}\tilde{u}|=n(x) \text { in } \Omega . \\ \tilde{u}(x)=\phi (x) \text { in } \partial \Omega , \end{array} \right. \end{aligned}$$

We let

$$\begin{aligned} \begin{aligned} L(x,y)=&\inf \left\{ \int _{0}^{T_{0}}n(\zeta (s))ds : (T_{0},\zeta ) \text { such that } \right. \\&\left. \zeta (0)=x,\zeta (T_{0})=y,\left| \frac{d\zeta }{ds}\right| \le {1} \text { a.e in } [0,T_{0}], \zeta (t)\in \bar{\Omega } \quad \forall {t}\in [0,T_{0}] \right\} . \end{aligned} \end{aligned}$$

One has a representation formula for solutions in terms of *L*.

#### Lemma 8

( Crandall and Lions 1984, Theorem 5.3) The viscosity solution to (20) is unique and given by

$$\begin{aligned} \tilde{u}(x)=\inf _{y\in \partial \Omega }[\phi (y)+L(x,y)]. \end{aligned}$$

It is straightforward to verify that if $\tilde{u}$ solves (20), then $u=-\tilde{u}$ solves

21 $$\begin{aligned} \left\{ \begin{array}{l} -|\partial _{x}u|=-n(x) \text { in } \Omega , \\ u(x)=-\phi (x) \text { in } \partial \Omega , \end{array} \right. \end{aligned}$$

and thus the solution to (18) is

$$\begin{aligned} u=\sup _{y\in \partial \Omega }[\phi '(y)+L'(x,y)], \end{aligned}$$

where $\phi '$ prescribes the boundary value and

22 $$\begin{aligned} \begin{aligned} L'(x,y)=&\sup \left\{ -\int _{0}^{T_{0}}\sqrt{a_M-a({\zeta (s)})}ds : (T_{0},\zeta ) \text { such that } \right. \\&\left. \zeta (0)=x,\zeta (T_{0})=y,\left| \frac{d\zeta }{ds}\right| \le {1} \text { a.e in } [0,T_{0}], \zeta (t)\in \bar{\Omega } \quad \forall {t}\in [0,T_{0}] \right\} . \end{aligned} \end{aligned}$$

When a general *n*(*x*) in (20) vanishes at multiple points in $\Omega$, then one cannot guarantee a unique viscosity solution, as is the case for $n(x)=\sqrt{a_{M}-a(x)}$. However, the representation formula still applies between the zeros of *n*(*x*) and this is enough to refine the set of concentration points.

We are now ready to prove Lemma 4 which is the first part of Theorem 1.

#### Proof of Lemma 4

If *u*(*x*) does not intersect $\frac{cx}{2}$ between the two maxima $x_i$ and $x_{i+1}$ then the constraint $\max {}_{x\in \mathbb {R}}u(x)-\frac{cx}{2}=0$ then implies that $u(x)<\frac{cx}{2}$ for $x\in [x_{i},x_{i+1}]$. In this case $p_\varepsilon$ converges to zero in $(x_{i},x_{i+1})$ and we are done. Instead, we suppose $x^{*}$ is an intersection point between *u*(*x*) and $\frac{cx}{2}$, and define $x_{i}^{+}$ as the minimum solution to $a(x)=a_{M}-\frac{c^2}{4}$ such that $x_{i}^{+}>x_i$ and $x_{i+1}^{-}$ as the maximum solution to the same equation such that $x_{i+1}^{-}<x_{i+1}$. These are the only solutions on the interval $(x_i,x_{i+1})$ according to (A5). Furthermore $x^{*}\in \{x_i^{+},x_{i+1}^{-}\}\subset (x_i,x_{i+1})$ as a consequence of Corollary 1. Our aim is to show that $x^{*}=x_{i+1}^{-}$ (which is $\bar{x}_{i+1}$).

Similarly to Iglesias and Mirrahimi (2021) we apply the representation formula (22) between the two maxima $x_{{i}}$ and $x_{{i+1}}$ to get

$$\begin{aligned} u(x)=\max \left\{ f_1(x);f_2(x)\right\} , \end{aligned}$$

where $$f_1(x)=u(x_{{i}})-\int _{x_{{i}}}^{x}\sqrt{a_{M}-a(y)}dy$$ and $$f_2(x)=u(x_{{i+1}})-\int _{x}^{x_{{i+1}}}\sqrt{a_{M}-a(y)}dy$$.

To understand the geometric picture better we note that $f_1(x)$ is decreasing, and $f_2(x)$ is increasing. Therefore, we have the alternatives: a) $f_1(x)$ and $f_2(x)$ intersect at exactly one point $z\in (x_{i},x_{i+1})$, b) $u(x)=f_1(x)$ for $x\in [x_{i},x_{i+1}]$, c) $u(x)=f_2(x)$ for $x\in [x_{i},x_{i+1}]$. In fact, case (b) may be excluded since we have assumed there is an intersection of *u*(*x*) and $\frac{cx}{2}$ in the interval $(x_{i},x_{i+1})$ but in case (b) the only possible intersection is at $x_i$.

In the remaining cases (a) or (c), we see that the intersection of *u* with $\frac{cx}{2}$ cannot be at the point *z* where $f_1(z)=f_2(z)$ since either $z{=x_{i}}$ if (c) holds, or, if (a) holds, then *u*(*x*) would intersect $\frac{cx}{2}$ from above, since *u*(*x*) is decreasing for $x<z$. This contradicts the constraint.

Therefore, $x^{*}\in {}(z,x_{{i+1}})$ and we have, in this interval,

$$\begin{aligned} u(x)=u(x_{{i+1}})-\int _{x}^{x_{{i+1}}}\sqrt{a_{M}-a(y)}dy \end{aligned}$$

and

23 $$\begin{aligned} \partial _{x}u=\sqrt{a_{M}-a(x)}. \end{aligned}$$

Differentiating (23) we find

$$\begin{aligned} \partial _{xx}u=-\frac{1}{2}\frac{a'(x)}{\sqrt{a_M-a(x)}}. \end{aligned}$$

We must also have that $\partial _{xx}u(x^{*})<0$ since otherwise *u* will exceed $\frac{cx}{2}$. By the definition of the points $x_{{i}}^{+}$ and $x_{{i+1}}^{-}$ we have that $a'(x_{{i}}^{+})<0$ and $a'(x_{{i+1}}^{-})>0$, therefore only $x_{{i+1}}^{-}$ is a possible concentration point. $\square$

We can now relate this to the solution $n_\varepsilon (x,t)$ of (2), which we do with Lemma 5. Firstly, we will need the following lemma.

#### Lemma 9

Assume $\lambda _\varepsilon <0$. Then exists an integrable function $\overline{W}_\varepsilon (x)$ such that

$$\begin{aligned} {N_\varepsilon (x,t)e^{\int _{0}^{t}\rho _{\varepsilon }(s)ds+\lambda _{\varepsilon }t}}\le \overline{W}_\varepsilon (x),~~\forall x\in \mathbb {R} \end{aligned}$$

#### Proof

Let $W_\varepsilon (x,t)=N_\varepsilon (x,t)e^{\int _{0}^{t}\rho _{\varepsilon }(s)ds+\lambda _{\varepsilon }t}$ which solves

24 $$\begin{aligned} {\left\{ \begin{array}{ll} \partial _{t}W_\varepsilon -\mathcal {L}W_\varepsilon =0,& ~~(x,t)\in {}\mathbb {R}\times (0,\infty )\\ W_\varepsilon (x,0)=n_0(x),& ~~x\in \mathbb {R}, \end{array}\right. } \end{aligned}$$

where $\mathcal {L}w:=c\varepsilon \partial _{x}w+\varepsilon ^2\partial _{xx}w+(a(x)+\lambda _\varepsilon )w$. We also let $\eta {>0}$ be a small constant. Then $u_\eta (x,t)=W_\varepsilon (x,t)e^{\eta {t}}$ solves

$$\begin{aligned} \partial _{t}u_\eta -\varepsilon c\partial _{x}u_\eta -\varepsilon ^2\partial _{xx}u_\eta =u_\eta \left( a(x)+\lambda _\varepsilon +\eta \right) . \end{aligned}$$

The function $\phi _\eta (x,t)=e^{\eta {t}}p_\varepsilon (x)$ is a positive entire solution of this differential equation that satisfies hypothesis (C2) as in Appendix A.3. We thus apply Corollary 2 in Appendix A.3 and findThe $e^{\eta {t}}$ terms cancel, so that . Thus there is a $T_1$ such that $W(x,t)<2\alpha _\varepsilon {}p_\varepsilon (x)$ for all $(x,t)\in {}[-R_0,R_0]\times {}[T_1,\infty )$. We next claim that $W_\varepsilon (x,t)\le {}\tilde{W}_\varepsilon (x,t)=e^{C_1-C_2|x|+(a_{M}+\lambda _\varepsilon )t}$ for all $(x,t)\in {}\mathbb {R}\times {}[0,\infty )$. Substituting $\tilde{W}_\varepsilon (x,t)$ into the differential equation gives

$$\begin{aligned} \partial _{t}\tilde{W}_\varepsilon -\mathcal {L}\tilde{W}_\varepsilon \ge {}-\varepsilon ^{2}C_2^2+\varepsilon {}C_2-(a(x)-a_{M}), \end{aligned}$$

which is positive, for small enough $\varepsilon$, by the definition of $a_{M}$ as the maximum of *a*(*x*). Since $n_0(x)\le {}e^{C_1-C_2|x|}$ by assumption (A3), the comparison theorem yields the desired result.

We define $\overline{W}_{{\varepsilon }}(x)$ as

$$\overline{W}_\varepsilon (x)={\left\{ \begin{array}{ll} e^{C_3+C_1-C_2|x|+(a_{1,M}+\lambda _\varepsilon )T_1}~~& x<-R_0,\\ 2\alpha _\varepsilon p_\varepsilon (x)~~& |x|<R_0,\\ e^{C_4+C_1-C_2|x|+(a_{1,M}+\lambda _\varepsilon )T_1}~~& x>R_0, \end{array}\right. }$$

where we pick $C_3$ and $C_4$ to ensure that $\overline{W}_\varepsilon (\pm {}R_0)=2\alpha _\varepsilon {}p_\varepsilon (\pm {}R_0)$. By construction $\overline{W}_\varepsilon$ dominates $W_\varepsilon (x,t)$ on the parabolic boundary of $((-\infty ,-R_0)\cup {}(R_0,\infty ))\times {}(T_1,\infty )$. We also check that

$$\begin{aligned} \partial _{t}\overline{W}_\varepsilon -\mathcal {L}\overline{W}_\varepsilon \ge {}-\varepsilon ^{2}C_2^2+\varepsilon {}C_2-(-{d}+\lambda _\varepsilon ), \end{aligned}$$

which is positive for $\varepsilon$ small enough. Thus by an application of the comparison theorem, $W_\varepsilon (x,t)\le {}\overline{W}_\varepsilon {(x)}$ for $(x,t)\in ((-\infty ,-R_0)\cup {}(R_0,\infty ))\times {}(0,\infty )$. In particular $W_\varepsilon (x,t)$ is bounded by a constant in time integrable function. $\square$

We can now proceed with the proof of Lemma 5.

#### Proof of Lemma 5

The first part follows similarly to the proof of proposition 2 in Iglesias and Mirrahimi (2018). We again let $\eta {>0}$ be a small constant, and now let ${\hat{u}_\eta (x,t)}=N_\varepsilon (x,t)e^{\frac{cx}{2\varepsilon }+\int _{0}^{t}\rho _{\varepsilon }(s)ds+\lambda _{\varepsilon }t+\eta {t}}$ which solves

$$\begin{aligned} \partial _{t}u_\eta -\varepsilon ^2\partial _{xx}u_\eta =u_\eta \left( a(x)-\frac{c^2}{4}+\lambda _\varepsilon +\eta \right) . \end{aligned}$$

The function $\phi _\eta (x,t)=e^{\eta {t}}{\hat{p}_\varepsilon }(x)$ is a positive entire solution to the differential equation (without initial conditions) that satisfies hypothesis (C2) as in Appendix A.3. We thus apply Corollary 2, and find, for a constant $\alpha _\varepsilon >0$ thatThe $e^{\eta {t}}$ terms cancel so we obtain:Moreover, this convergence is exponential. Recalling that $\hat{p}_\varepsilon =p_\varepsilon {}e^\frac{cc}{2\varepsilon }$ we may rewrite it in terms of ${p}_\varepsilon$ (up to a change in the constant $\alpha _\varepsilon$):This implies

25 $$\begin{aligned} N_\varepsilon (x,t)e^{\int _{0}^{t}\rho _{\varepsilon }(s)ds+\lambda _{\varepsilon }t}=\alpha _{\varepsilon }{p}_\varepsilon {}+\Sigma _{1}(x,t)e^{-\frac{cx}{2\varepsilon }}, \end{aligned}$$

where  exponentially. We use this to write

$$\begin{aligned} \frac{N_\varepsilon }{\rho _{\varepsilon }}=\frac{\alpha _\varepsilon {p}_\varepsilon +\Sigma _{1}(x,t)e^{-\frac{cx}{2\varepsilon }}}{\int \alpha _\varepsilon {p}_\varepsilon +\Sigma _{1}(y,t)e^{-\frac{cx}{2\varepsilon }}dy}, \end{aligned}$$

and

$$\begin{aligned} \rho _{\varepsilon }(t)=\int _{\mathbb {R}}\alpha _\varepsilon {p}_\varepsilon dy+\int _{\mathbb {R}}\Sigma _{1}(y,t)e^{-\frac{cx}{2\varepsilon }}dy. \end{aligned}$$

We need to show that the latter term converges to 0 as $t\rightarrow {\infty }$. By Lemma 9 and (25)

$$\begin{aligned} {\left| \Sigma _{1}(x,t)\right| }e^{-\frac{cx}{2\varepsilon }}\le \overline{W}_\varepsilon (x)+\alpha _\varepsilon p_\varepsilon , \end{aligned}$$

and each term on the right-hand side is integrable. Thus by the Dominated Convergence Theorem, using the exponential decay of $\Sigma _1(x,t)$ in time, we have .

This completes the proof. $\square$

We can now prove Lemma 6.

#### Proof of Lemma 6

This follows similarly to Iglesias and Mirrahimi (2018). Computations identical to the preceding lemma show thatThis convergence is exponential. By integrating (4), we obtain:

$$\begin{aligned} \frac{d\rho _{\varepsilon }}{dt}=\rho _{\varepsilon }\left( \int _{{\mathbb {R}}}a(y){p}_\varepsilon (y){dy}+\Sigma _{2}(t)-\rho _{\varepsilon }\right) , \end{aligned}$$

where $\Sigma _{2}(t)$ is exponentially decreasing. By applying Gronwall’s lemma, we show the long time limit of $\rho _{\varepsilon }$ is $\int _{{\mathbb {R}}}a(y){p}_\varepsilon (y)$. This can be done as follows:

Firstly, we claim $\rho _{\varepsilon }(t)$ is eventually bounded above by $\overline{\gamma }=\int _{{\mathbb {R}}}a(y){p}_\varepsilon (y)dy+2|\Sigma _{2}(T)|$ for any fixed *T*. Suppose that $\rho _{\varepsilon }(t)$ exceeds $\overline{\gamma }$ at some point $t_{1}>T$ and let $t_{2}$ be next time where $\rho _{\varepsilon }(t)=\overline{\gamma }$ if such a point exists and be $\infty$ otherwise. Then for all $t\in (t_{1},t_{2})$

$$\begin{aligned} \frac{d\rho _{\varepsilon }}{dt}&\le {}\rho _{\varepsilon }\left( \Sigma _{2}(t)-2|\Sigma _{2}(T)|\right) \\&\le {}-\rho _{\varepsilon }|\Sigma _{2}(T)|. \end{aligned}$$

Thus by Gronwall’s inequality,

$$\begin{aligned} \rho _{\varepsilon }(t)\le \rho _{\varepsilon }(t_{1})e^{-(t-t_{1})|\Sigma _2(T)|}. \end{aligned}$$

This implies $t_{2}$ is finite. But now it is clear $\rho _{\varepsilon }'(t)$ will be negative if $\rho _{\varepsilon }$ exceeds $\overline{\gamma }$ again, thus $\overline{\gamma }$ is an upper bound for all $t>t_{1}$. Since this is true for any $T>0$ this shows:

$$\begin{aligned} \limsup _{t\rightarrow \infty }\rho _{\varepsilon }(t)=\int _{{\mathbb {R}}}a(y){p}_\varepsilon (y){dy}. \end{aligned}$$

We must still find a lower bound. Firstly, since $N\ge {0}$ we can ensure 0 is a lower bound for $\rho _{\varepsilon }(t)$. We define $\hat{\rho _{\varepsilon }}(t)=\rho _{\varepsilon }(t)e^{-\int _{0}^{t}\Sigma _2(s)ds}$ which solves:

$$\begin{aligned} \frac{d\hat{\rho _{\varepsilon }}}{dt}=\hat{\rho _{\varepsilon }}\left( \int _{{\mathbb {R}}}a(y){p}_\varepsilon (y){dy}-\hat{\rho _{\varepsilon }}e^{\int _{0}^{t}\Sigma (s)ds}\right) . \end{aligned}$$

The term $e^{\int _{0}^{t}\Sigma _2(s)ds}$ is bounded above by $k=e^{\int _{0}^{\infty }|\Sigma _2(s)|ds}$ so we have:

$$\begin{aligned} \frac{d\hat{\rho _{\varepsilon }}}{dt}\ge \hat{\rho _{\varepsilon }}\left( \int _{{\mathbb {R}}}a(y){p}_\varepsilon (y){dy}-k\hat{\rho _{\varepsilon }}\right) . \end{aligned}$$

This means that $\frac{d\hat{\rho _{\varepsilon }}}{dt}$ is positive as long as $\hat{\rho _{\varepsilon }}\le {}\frac{\int _{{\mathbb {R}}}a(y){p}_\varepsilon (y){dy}{}}{k}$. In particular this means $\hat{\rho _{\varepsilon }}$ is increasing and thus $\rho _{\varepsilon }$ has:

$$\begin{aligned} \liminf _{t\rightarrow \infty }\rho _{\varepsilon }\ge \rho _\text {min}>0. \end{aligned}$$

The lower bound is important in what follows. Proceeding similarly as before we define $\underline{\gamma }=\int _{{\mathbb {R}}}a(y){p}_\varepsilon (y){dy}-2|\Sigma _{2}(T)|$ for an arbitrary *T*. We suppose there is a time $t_{3}>T$ such that $\int _{{\mathbb {R}}}a(y){p}_\varepsilon (y)+\Sigma _{2}(t_{3})>0$ and $\frac{\rho _\text {min}}{2}<\rho _{\varepsilon }(t)<\underline{\gamma }$ for all $t\in (t_3,t_4)$ where $t_4$ is the next time such that $\rho _\varepsilon =\underline{\gamma }$ or $\infty$ if no such time exists.

Then:

$$\begin{aligned} \frac{d\rho _{\varepsilon }}{dt}\ge \frac{\rho _\text {min}}{2}\left( \int a(x)p_\varepsilon -|\Sigma _2(T)|-\rho _{\varepsilon }\right) . \end{aligned}$$

Let $\tilde{\rho _{\varepsilon }}=\int _{{\mathbb {R}}}a(y){p}_\varepsilon (y){dy}-|\Sigma _2(T)|-\rho _{\varepsilon }$. Then $\tilde{\rho _{\varepsilon }}$ satisfies:

$$\begin{aligned} \frac{d\tilde{\rho _{\varepsilon }}}{dt}\le -\frac{\rho _\text {min}}{2}\tilde{\rho _{\varepsilon }}. \end{aligned}$$

Similarly to before, $\tilde{p}_\varepsilon$ is decreasing (and thus $\rho _{\varepsilon }$ is increasing) at an exponential rate in this case and therefore $t_4$ is finite. But $\frac{d\rho _\varepsilon }{dt}(t)>0$ for any $t>t_4$ such that $\rho _\varepsilon (t)=\underline{\gamma }$ and so cannot decrease below $\underline{\gamma }$ again. This gives:

$$\begin{aligned} \liminf _{t\rightarrow \infty }\rho _{\varepsilon }(t)=\int _{{\mathbb {R}}}a(y){p}_\varepsilon (y){dy}. \end{aligned}$$

$$\square $$

#### Proof of Theorem 1

Theorem 1 follows by combining Lemmas 4 to 6. $\square$

### Proof of Theorem 3

For this section, we prove the main theorem concerning the solution of (11), and we recall that we suppress the $\varepsilon$ notation for the solutions to the transformed problems. In order to prove Theorem 3, we aim to determine local growth rates (in terms of the eigenvalues $\lambda _{i,\varepsilon }$) of solutions.

#### Lemma 10

For each $i\in {1,2}$ let $p_{i,\varepsilon }^{\infty }(x)$ be the $L_\infty (\mathbb {R})$ normalized solution to (12). We have the following bounds:

$$\begin{aligned} e^{-\frac{\underline{\kappa }_{i}|x-x_\varepsilon |}{\varepsilon }}\le p_{i,\varepsilon }^{\infty }(x)\le \min \left\{ 1,e^{\frac{-\overline{\kappa }_{i}(|x|-R_0)}{\varepsilon }}\right\} ,~~x\in \mathbb {R}, \end{aligned}$$

where $x_\varepsilon$ is a point such that $p_{i,\varepsilon }^{\infty }(x_\varepsilon )=1$, $\underline{\kappa }_i=\frac{c_i}{2}{+2\sqrt{\left| a_m+\lambda _{i,\varepsilon }-\frac{c_i^2}{4}\right| }}$ and $\overline{\kappa }_i=\frac{\sqrt{a_M}+c_i}{2}$. Moreover, we have that $\Vert {}p_{i,\varepsilon }\Vert _{L^\infty (\mathbb {R})}\le {}\frac{K_1}{\varepsilon }$, where $K_1$ is independent of $\varepsilon$.

#### Proof

This follows identically to the proof of Lemma 3. The $L^\infty$ bound on $p_{i,\varepsilon }$ follows from writing $p_{i,\varepsilon }=\frac{p_{i,\varepsilon }^\infty }{\Vert {p_{i,\varepsilon }^\infty }\Vert _{L^{1}(\mathbb {R})}}\le {}\frac{2}{\varepsilon {}\underline{\kappa _{i}}}$ where the inequality is a consequence of the lower bound on $p_{i,\varepsilon }^{\infty }$. $\square$

The next lemma is the main ingredient in the proof. To state it, we first let $\lambda _{i,0}=\lim _{\varepsilon \rightarrow {{0}}}\lambda _{i,\varepsilon }$ and define $\underline{i}\in \{1,2\}$ such that $\lambda _{\underline{i},0}=\min \{\lambda _{1,0},\lambda _{2,0}\}$. Note that we could define $\underline{i}$ in terms of $\lambda _{i,\varepsilon }$ instead, and these definitions would coincide for sufficiently small $\varepsilon$ provided $\lambda _{1,0}\ne {}\lambda _{2,0}$. We also let $\overline{i}\in \{1,2\}$ be such that $\lambda _{\overline{i},0}=\max \{\lambda _{1,0},\lambda _{2,0}\}.$

We define $W_{\underline{i}}(x,t):=N_{\underline{i}}(x,t)e^{\int _{0}^{t}\rho (s)ds+\lambda _{\underline{i},\varepsilon }{t}}$ which solves

26 $$\begin{aligned} {\left\{ \begin{array}{ll} \partial _{t}W_{\underline{i}}-\mathcal {L}_{\underline{i}}W_{\underline{i}}=0, \\ W_{\underline{i}}(x,0)=n_0(x), \end{array}\right. } \end{aligned}$$

where $\mathcal {L}_{\underline{i}}w:=\varepsilon {}c_{\underline{i}}\partial _{x}w+\varepsilon ^2\partial _{xx}w+w(a_{\underline{i}}(x)+a_{\overline{i}}(x+(-1)^{\underline{i}}\varepsilon {}Ct)-{d}+\lambda _{\underline{i},\varepsilon })$. Our aim is to establish that $W_{\underline{i}}(x,t)$ decays exponentially in a ball that contains the support of $a_{\overline{i}}(x-\varepsilon {}Ct)$. The main result will follow from the bounds we obtain as a corollary of this result.

#### Lemma 11

We assume (B1)-(B5), that $\lambda _{1,0}\ne {}\lambda _{2,0}$, and that $\lambda _{\underline{i},0}<0$ . Then

$$\begin{aligned} W_{\underline{i}}(x,t)\le e^{\frac{K}{\varepsilon }-\left( t-\frac{\gamma }{\varepsilon }\right) {\eta }} \end{aligned}$$

for $(x,t)\in {}[-R_0+(-1)^{\overline{i}}\varepsilon {C}t,R_0+\varepsilon {}(-1)^{\overline{i}}Ct]\times \left( \frac{\gamma }{\varepsilon },\infty \right)$ where the positive constants *K*, $\gamma$, and $\eta$ are independent of $\varepsilon$.

Before providing the details of the proof, we motivate the construction of a supersolution of the form $\overline{W}(x,t)=e^{\frac{1}{\varepsilon }\overline{\phi }(x,t)}$. In order to unburden the notation, we assume for now, without loss of generality that $\underline{i}=1$, since otherwise we apply the transformation $x\rightarrow {-x}$ and swap labels. Observe that

$$\begin{aligned} \partial _{t}\overline{W}-\mathcal {L}_1\overline{W}&=\overline{W}\left( \frac{1}{\varepsilon }\partial _{t}\overline{\phi }-\varepsilon {}\partial _{xx}\overline{\phi }-|\partial _{x}\overline{\phi }|^2-c_1\partial _{x}\overline{\phi }-(a_1(x)+a_2(x-\varepsilon {C}t)-{d}+\lambda _{1,\varepsilon })\right) ,\\&=\overline{W}\left( \frac{1}{\varepsilon }\partial _{t}\overline{\phi }-\varepsilon {}\partial _{xx}\overline{\phi }-\left| \partial _{x}\overline{\phi }+\frac{c_1}{2}\right| ^2-\left( a_1(x)+a_2(x-\varepsilon {C}t)-{d}+\lambda _{1,\varepsilon }-\frac{c_1^2}{4}\right) \right) , \end{aligned}$$

and therefore $\partial _{t}\overline{W}-\mathcal {L}_1\overline{W}\ge {0}$ if and only if

$$\begin{aligned} \frac{1}{\varepsilon }\partial _{t}\overline{\phi }-\varepsilon \partial _{xx}\overline{\phi }-\left| \partial _{x}\overline{\phi }+\frac{c_1}{2}\right| ^2-\left( a_1(x)+a_2(x-\varepsilon {C}t)-{d}+\lambda _{1,\varepsilon }-\frac{c_1^2}{4}\right) \ge {0}. \end{aligned}$$

Therefore, to establish that $\overline{W}(x,t)$ is a generalised supersolution to (26) (as defined in Section A) , we require that:

1. $$\overline{\phi }\in {}C^{}(\mathbb {R}\times (0,\infty ))$$.
2. The differential inequality $\frac{1}{\varepsilon }\partial _{t}\overline{\phi }-\varepsilon {}\partial _{xx}\overline{\phi }-\left| \partial _{x}\overline{\phi }+\frac{c_1}{2}\right| ^2-\left( a_1(x)+a_2(x-\varepsilon {C}t)-{d}+\lambda _{1,\varepsilon }-\frac{c_1^2}{4}\right) \ge {0}$ is satisfied in the classical sense wherever $\overline{\phi }$ is sufficiently smooth.
3. At each point $(x_0,t_0)$ where $\overline{\phi }$ fails to be $C^{2,1}(\mathbb {R}\times (0,\infty ))$ there exists an open neighbourhood *U* containing $(x_0,t_0)$ and a function $\tilde{w}\in {}C^{2,1}(U)$ such that $\tilde{w}(x,t)\ge {}\overline{\phi }(x,t)$ for $(x,t)\in {}U$, $\tilde{w}(x_0,t_0)=\overline{\phi }(x_0,t_0)$, and $\tilde{w}$ satisfies $\frac{1}{\varepsilon }\partial _{t}\tilde{w}-\varepsilon {}\partial _{xx}\tilde{w}-|\partial _{x}\tilde{w}|^2-\left| \partial _{x}\tilde{w}+\frac{c_1}{2}\right| ^2-\left( a_1(x)+a_2(x-\varepsilon {C}t)-{d}+\lambda _{1,\varepsilon }-\frac{c_1^2}{4}\right) \ge {0}$ in the classical sense.

The condition (C3) is satisfied at a point $x_b$ if $\overline{\phi }$ is locally the minimum of two functions which satisfy the differential inequality in the classical sense in a neighbourhood of that point. To locally be the minimum of such functions requires that the left derivative is greater than the right derivative, i.e. $\partial _{x}\overline{\phi }(x_b{-},t)>\partial _{x}\overline{\phi }(x_b{+},t)$. To check that (C3) holds at $x_b$, it is enough to check this condition and that there is a small neighbourhood of $x_b$ such that each piece of the $\overline{\phi }$ can be extended smoothly across the point, which in particular will be true if the differential inequality (C2) is not strict at $x_b$.

We now define $\overline{\phi }(x,t)$ as

27 $$\begin{aligned} &  \overline{\phi }(x,t)=\min \left\{ u_1(x)+\varepsilon \nu \left( t-\frac{\gamma }{\varepsilon }\right) ,u_2(x-\varepsilon {C}t)-\varepsilon \nu \left( t-\frac{\gamma }{\varepsilon }\right) \right\} \nonumber \\ &  \quad -\min \{R_0c_1,-R_0c_2\}{-\cos (\xi )}+\tilde{K}, \end{aligned}$$

where $\tilde{K}$, $\gamma$, and $\nu$ are positive constants to be defined during the proof, and $\xi$ is a positive constant that will be defined below. The functions $u_i(x)$, $i=1,2$, are given by

28 $$\begin{aligned} u_1(x)={\left\{ \begin{array}{ll} R_0c_1{-\cos (\xi )} & x\in (-\infty ,-2R_0{-\frac{\pi }{2\alpha _1}-\frac{\xi }{\alpha _1}}),\\ R_0c_1+\sin \left( \alpha _1(x+2R_0)\right) & x\in [-2R_0{-\frac{\pi }{2\alpha _1}-\frac{\xi }{\alpha _1}},-2R_0],\\ -\frac{c_1x}{2} & x\in [-2R_0,\infty ], \end{array}\right. } \end{aligned}$$

where $$\alpha _1=\frac{\sqrt{2a_{1,M}}-c_1}{2}$$, and

29 $$\begin{aligned} u_2(x)={\left\{ \begin{array}{ll} -\frac{c_2x}{2} & x\in (-\infty ,2R_0],\\ -R_0c_2+\sin \left( \alpha _2(2R_0-x)\right) & x\in [2R_0,2R_0{+\frac{\pi }{2\alpha _2}+\frac{\xi }{\alpha _2}}],\\ -R_0c_2{-\cos (\xi )} & x\in [2R_0{+\frac{\pi }{2\alpha _2}+\frac{\xi }{\alpha _2}},\infty ), \end{array}\right. } \end{aligned}$$

where $\alpha _2=\frac{\sqrt{2a_{2,M}}+c_2}{2}$. The positive constant $\xi$ is chosen such that the following inequalities are satisfied:

30 $$\begin{aligned} \left| \alpha _1\sin (\xi )-\frac{c_1}{2}\right| ^2&<a_{1,M}, \end{aligned}$$

31 $$\begin{aligned} \left| \alpha _2\sin (\xi )+\frac{c_2}{2}\right| ^2&<\frac{1}{2}\left( a_{1,M}-\frac{c_1^2}{4}\right) +\frac{c_2^2}{4}. \end{aligned}$$

Such a choice is possible because of the continuity of the LHS and the fact that the strict inequalities are satisfied for $\xi =0$ (because have assumed $a_{1,M}>\frac{c_1^2}{4}$). We also assume $\xi <\frac{\pi }{2}$.

**Fig. 3:**
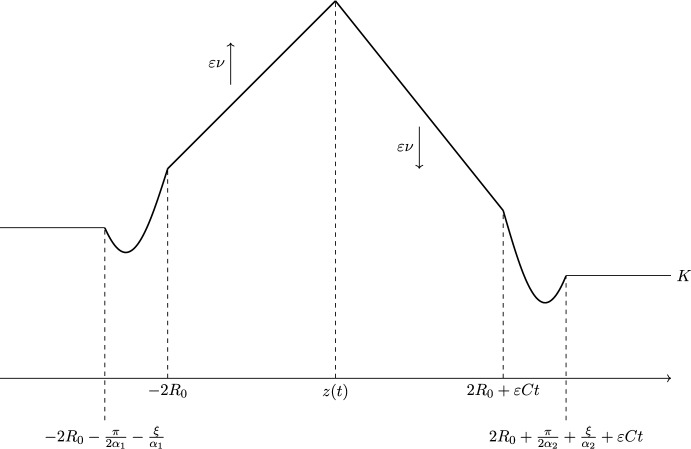
A diagram illustrating $\overline{\phi }(x,t)$ (for *t* sufficiently large) where $\overline{W}(x,t)=e^{\frac{1}{\varepsilon }\overline{\phi }(x,t)}$ is the generalised supersolution to (26). Here *z*(*t*) is the intersection between the $u_1(x)+\varepsilon \nu \left( t-\frac{\gamma }{\varepsilon }\right)$ and $u_2(x-\varepsilon {C}t)-\varepsilon \nu {}\left( t-\frac{\gamma }{\varepsilon }\right)$ assumed to be in the interval $[-2R_0,2R_0+\varepsilon {C}t]$, where $u_1$ and $u_2$ are defined by (28) and (29) respectively

If the intersection *z*(*t*) between $u_1(x)+\varepsilon {}\nu \left( t-\frac{\gamma }{\varepsilon }\right)$ and $u_2(x-\varepsilon {C}t)-\varepsilon \nu \left( t-\frac{\gamma }{\varepsilon }\right)$ occurs on the interval $[-2R_0,2R_0+\varepsilon {C}t]$ then the intersection is between the linear parts of each function. In this case, *z*(*t*) satisfies

$$\begin{aligned} -\frac{c_1}{2}z(t)+\varepsilon \nu \left( t-\frac{\gamma }{\varepsilon }\right) =-\frac{c_2}{2}(z(t)-\varepsilon Ct)-\varepsilon \nu \left( t-\frac{\gamma }{\varepsilon }\right) , \end{aligned}$$

which can be solved to obtain

$$\begin{aligned} z(t)=c_2\gamma +\frac{2\varepsilon }{C}\left( \frac{c_2}{2}C-2\nu \right) \left( t-\frac{\gamma }{\varepsilon }\right) . \end{aligned}$$

We note that if the RHS defined by the above formula lies in the interval $[-2R_0,2R_0+\varepsilon {C}t]$, and *t* is large enough that the supports of $a_1(x)$ and $a_2(x-\varepsilon {C}t)$ do not intersect, then in fact *z*(*t*) is given by the RHS.

We illustrate $\overline{\phi }(x,t)$ in Fig. 3 for the case where $z(t)\in [-2R_0,2R_0+\varepsilon {C}t]$ . The essential idea behind the construction is that the linear parts control the solution in regions containing the supports of $a_1(x)$ and $a_2(x-\varepsilon {C}t)$, the constant pieces ensure we can pick a *K* such that $W_1(x,t^{*})\le {}\overline{W}(x,t^{*})$ for some sufficiently large time $t^{*}$, and the trigonometric pieces connect the linear and constants pieces and enable us to satisfy condition (C3) at each non-smooth point.

#### Proof of Lemma 11

We show that conditions (C1)-(C3) hold for $\overline{\phi }(x,t)$. The continuity of $\overline{\phi }$ is clear by the construction. We check that (C2) and (C3) hold for $\overline{u}_1(x)+\nu \left( t-\frac{\gamma }{\varepsilon }\right)$. Where $u_1(x)$ is constant (e.g. for $x\in (-\infty ,-2R_0{-\frac{\pi }{2\alpha _1}-\frac{\xi }{\alpha _1}})$) the differential inequality becomes

32 $$\begin{aligned} \nu -\lambda _{1,\varepsilon }+{d}\ge {0} \end{aligned}$$

where we have used the fact the supports of $a_1(x)$ and $a_2(x-\varepsilon {C}t)$ do not overlap with this region. This inequality is satisfied trivially, for sufficiently small $\varepsilon$ since we have assumed $\lambda _{1,0}<0$. Similar computations for $x\in$ $(2R_0{+\frac{\pi }{2\alpha _1}+\frac{\xi }{\alpha _1}},\infty )$ yield the requirement that

33 $$\begin{aligned} -\nu -\lambda _{1,\varepsilon }+{d}\ge {0}. \end{aligned}$$

We now focus the linear regions. Firstly, we assume $t>\frac{\gamma }{\varepsilon }$ and $\gamma >\frac{2R_0}{C}$ so that the supports of $a_1(x)$ and $a_2(x-\varepsilon {C}t)$ do not overlap. We now obtain conditions to ensure that $z(t)\in [-2R_0,2R_0+\varepsilon {C}t]$ for all $t\ge {}\frac{\gamma }{\varepsilon }$. This is the case at $t=\frac{\gamma }{\varepsilon }$ since the inequality to be satisfied is:

$$\begin{aligned} -2R_0<c_2\gamma <2R_0+C\gamma , \end{aligned}$$

and $C>c_2>0$. This is the case for all $t>\frac{\gamma }{\varepsilon }$ provided that

$$\begin{aligned} 0<\frac{2\varepsilon }{C}\left( \frac{c_2}{2}C-2\nu \right) t<C\varepsilon {t}. \end{aligned}$$

The upper bound is trivial since $c_2<C$ and $\nu >0$, and so we only need to satisfy

34 $$\begin{aligned} 0<\frac{c_2}{2}C-2\nu . \end{aligned}$$

Assuming that $z(t)\in [-2R_0,2R_0+\varepsilon {C}t]$ (as will be clear once we finalise the choice for $\nu$) condition (C2) in the region $(-2R_0,z(t))$ is

$$\begin{aligned} \nu -\left( a_1(x)-{d}+\lambda _{1,\varepsilon }-\frac{c_1^2}{4}\right) \ge 0. \end{aligned}$$

This condition is satisfied for any $\nu >0$ independent of $\varepsilon$, for $\varepsilon$ small enough, since by Lemma 2 we have that  which implies the limit of the LHS is $\nu +a_{1,M}-a_1(x)>\nu$. An identical computation in the region $[z(t),2R_0+\varepsilon {C}t]$, where $\overline{\phi }=u_2(x-\varepsilon {C}t)-\varepsilon \nu \left( t-\frac{\gamma }{\varepsilon }\right) -\min \{R_0c_1,-R_0c_2\}+\tilde{K}$, yields the condition

$$\begin{aligned} -\nu -\left( a_2(x-\varepsilon {C}t)-{d}+\lambda _{1,\varepsilon }-\frac{c_2^2}{4}\right) \ge {0}. \end{aligned}$$

Using the bound $a_{2}(x)\le {}a_{2,M}$ and the convergence  we obtain the sufficient condition (for $\varepsilon$ small enough)

35 $$\begin{aligned} -\nu -(\lambda _{1,0}-\lambda _{2,0})>0. \end{aligned}$$

At this point we observe that (35) implies (33) for sufficiently small $\varepsilon$.

To satisfy (C2) for $x{}\in \left( -2R_0{-\frac{\pi }{2\alpha _1}-\frac{\xi }{\alpha _1}},-2R_0\right)$ we require

36 $$\begin{aligned} \nu -\varepsilon \alpha _1^2\sin (\alpha _1(x+2R_0))-\left| \alpha _1\cos (\alpha _1(x+2R_0))+\frac{c_1}{2}\right| ^2-\left( \lambda _{1,\varepsilon }-\frac{c_1^2}{4}-{d}\right) \ge {0}. \nonumber \\ \end{aligned}$$

Since $\cos (\alpha _{1}(x+2R_0))$ is increasing for $x\in [-2R_0{-\frac{\pi }{2\alpha _1}-\frac{\xi }{\alpha _1}},-2R_0]$ (provided $\xi <\frac{\pi }{2}$) the extreme points of $\left| \alpha _1\cos (\alpha _1(x+2R_0))+\frac{c_1}{2}\right| ^2$ occur at the end points of the interval. Therefore, we have that $\left| \alpha _1\cos (\alpha _1(x+2R_0))+\frac{c_1}{2}\right| ^2<\max \left\{ \left| \alpha _{1}\sin (\xi )-\frac{c_1}{2}\right| ^2,\frac{a_{1,M}}{2}\right\}$ for $x\in [-2R_0{-\frac{\pi }{2\alpha _1}-\frac{\xi }{\alpha _1}},-2R_0]$

By taking the limit as $\varepsilon \rightarrow {0}$, we see that (36) is satisfied for small enough $\varepsilon$ provided that

$$\begin{aligned} \nu -{\max \left\{ \left| \alpha _{1}\sin (\xi )-\frac{c_1}{2}\right| ^2,\frac{a_{1,M}}{2}\right\} }+a_{1,M}>0, \end{aligned}$$

where we have again used the fact that  to take the limit. The inequality then follows from (30).

To satisfy (C2) for $x\in (2R_0+\varepsilon {C}t,2R_0+\frac{\pi }{2\alpha _2}+\frac{\xi }{\alpha _2} +\varepsilon {C}t)$ similar calculations yield

$$\begin{aligned} -\nu -\varepsilon \alpha _2^2\sin (\alpha _2(2R_0-x))-\left| -\alpha _2\cos (\alpha _2(2R_0-x))+\frac{c_2}{2}\right| ^2-\left( \lambda _{1,\varepsilon }-\frac{c_2^2}{4}-{d}\right) \ge {0}, \end{aligned}$$

which follows (for sufficiently small $\varepsilon$) provided that

$$\begin{aligned} -\nu -\max \left\{ \left| \alpha _2\sin (\xi )+\frac{c_2}{2}\right| ^2,\frac{a_{2,M}}{2}\right\} +a_{1,M}-\frac{c_1^2}{4}+\frac{c_2^2}{4}>0. \end{aligned}$$

If $$\max \left\{ \left| \alpha _2\sin (\xi )+\frac{c_2}{2}\right| ^2,\frac{a_{2,M}}{2}\right\} =\frac{a_{2,M}}{2}$$ then

$$\begin{aligned} -\nu -\max \left\{ \left| \alpha _2\sin (\xi )+\frac{c_2}{2}\right| ^2,\frac{a_{2,M}}{2}\right\} +a_{1,M}-\frac{c_1^2}{4}+\frac{c_2^2}{4}&>-\nu +a_{1,M}-\frac{c_1^2}{4}-a_{2,M}+\frac{c_2^2}{4}+a_{2,M}\\&>-\nu -(\lambda _{1,0}-\lambda _{2,0}). \end{aligned}$$

The last term is positive given (35). If instead $\max \left\{ \left| \alpha _2\sin (\xi )+\frac{c_2}{2}\right| ^2,\frac{a_{2,M}}{2}\right\} {=}\left| \alpha _2\sin (\xi ) {+}\frac{c_2}{2}\right| ^2$ then by (31) we have

$$\begin{aligned} -\nu -\left| \alpha _2\sin (\xi )+\frac{c_2}{2}\right| ^2+a_{1,M}-\frac{c_1^2}{4}+\frac{c_2^2}{4}>-\nu +\frac{1}{2}\left( a_{1,M}-\frac{c_1^2}{4}\right) . \end{aligned}$$

Therefore a sufficient condition to satisfy (C2) for $x\in [2R_0+\varepsilon {C}t,2R_0+\frac{\pi }{2\alpha _2}+\frac{\xi }{\alpha _2} +\varepsilon {C}t]$ is

37 $$\begin{aligned} \nu <\frac{1}{2}\left( a_{1,M}-\frac{c_1^2}{4}\right) . \end{aligned}$$

We have now determined sufficient conditions on $\nu$ are (34), (35), and (37). We therefore choose $\nu =\frac{1}{2}\min \left\{ \frac{c_2C}{4},\lambda _{1,0}-\lambda _{2,0},{\frac{1}{2}\left( a_{1,M}-\frac{c_1^2}{4}\right) }\right\}$.

Next, we deal with the non-smooth points. At *z*(*t*), $-2R_0{-\frac{\pi }{2\alpha _1}-\frac{\xi }{\alpha _1}}$, and $2R_0{+\frac{\pi }{2\alpha _2}+\frac{ \delta }{\alpha _2}}+\varepsilon {}Ct$ this is trivial. At $x=-2R_0$ the condition (C3) is satisfied since

$$\begin{aligned} \partial _{x}\overline{\phi }(-2R_0{-},t)=\frac{\sqrt{2a_{1,M}}-c_1}{2}>-\frac{c_1}{2}=\partial _{x}\overline{\phi }(-2R_0{+},t). \end{aligned}$$

Similarly, at $x=2R_0+\varepsilon {C}t$, it is satisfied because

$$\begin{aligned} \partial _{x}\overline{\phi }((2R_0+\varepsilon {C}t)-,t)=-\frac{c_2}{2}>-\frac{c_2+\sqrt{2a_{2,M}}}{2}=\partial _{x}\overline{\phi }((2R_0+\varepsilon {C}t){+},t). \end{aligned}$$

Lastly, we need to pick $\gamma$ and *K* such that ${W}_1\left( x,\frac{\gamma }{\varepsilon }\right) \le {}e^{\varepsilon ^{-1}\overline{\phi }\left( x,\frac{\gamma }{\varepsilon }\right) }$ where $t=\frac{\gamma }{\varepsilon }$ is large enough so that the supports do not intersect. For $t>\frac{3R_0}{C\varepsilon }$ the supports of $a_1(x)$ and $a_2(x-\varepsilon {C}t)$ are separated by an interval of length at least $R_0$, so we take $\gamma =\frac{3R_0}{C}$. We then observe that by the comparison theorem, and the fact that from (A1) we have $a_1(x)+a_2(x-\varepsilon {C}t)-{d}\le {}a_{1,M}+a_{2,M}$, we have that $W_1(x,t)\le {}e^{C_1-C_2|x|+(a_{1,M}+a_{2,M}-\lambda _{1,\varepsilon })t}$ where we recall $C_1$ and $C_2$ from assumption (B3). Therefore, by taking $\tilde{K}=2(a_{1,M}+a_{2,M}-\lambda _{1,0})\gamma$, we obtain the desired inequality at $t=\frac{\gamma }{\varepsilon }$.

The lemma now follows by applying the comparison theorem in Appendix A.2, and taking $\eta =\nu$, and $K=\max _{x\in [-R_0+\varepsilon {}Ct,R_0+\varepsilon {}Ct]}\overline{\phi }\left( x,\frac{\gamma }{\varepsilon }\right)$, which is $\tilde{K}-\min \{R_0c_1,-R_0c_2\}+\frac{c_2R_0}{2}{-\cos (\xi )}$. $\square$

#### Lemma 12

Under the same assumptions as in Lemma 11 there is a constant $\alpha _\varepsilon >0$ such that .

#### Proof

Again, for the duration of the proof, we may assume ${\underline{i}=1}$ without loss of generality. We consider $U(x,t)=W_1(x,t+T+\frac{\gamma }{\varepsilon })e^{-\frac{K}{\varepsilon }}$ which solves

38 $$\begin{aligned} {\left\{ \begin{array}{ll} \partial _{t}U-\mathcal {L}U-a_2(x-\varepsilon {}Ct-\gamma {}C-\varepsilon {}T)U=0~~(x,t)\in \mathbb {R}\times \left( 0,\infty \right) , \\ U\left( x,0\right) =W_1\left( x+T,\frac{\gamma }{\varepsilon }\right) e^{-\frac{K}{\varepsilon }},~~x\in \mathbb {R}, \end{array}\right. } \end{aligned}$$

where now $\mathcal {L}u:=-\varepsilon {}c_1\partial _{x}u-\varepsilon ^{2}\partial _{xx}u-(a_1(x)+\lambda _{1,\varepsilon })$. We note that $0<a_2(x-\varepsilon {}Ct-\gamma {}C-\varepsilon {}T)U\le {}a_{2,M}e^{-\eta {t}-\eta {}T}$ due to Lemma 11. Therefore, we have that $\tilde{U}(x,t)\le {}U(x,t)\le {}\tilde{U}(x,t)e^{a_{2,M}e^{-\eta {}T}\int _{0}^{t}e^{-\eta {s}}ds}$ for all $(x,t)\in {}\mathbb {R}\times \left( \frac{\gamma }{\varepsilon },\infty \right)$ where $\tilde{U}(x,t)$ solves

39 $$\begin{aligned} {\left\{ \begin{array}{ll} \partial _{t}\tilde{U}-\mathcal {L}\tilde{U}=0~~(x,t)\in \mathbb {R}\times \left( 0,\infty \right) , \\ U\left( x,0\right) =W_1\left( x,T+\frac{\gamma }{\varepsilon }\right) e^{-\frac{K}{\varepsilon }},~~x\in \mathbb {R}, \end{array}\right. } \end{aligned}$$

but this is exactly the eigenvalue problem in the case of a single shifting peak, and we therefore know that $\tilde{U}(x,t)=\alpha _{\varepsilon ,T}p_{1,\varepsilon }(x,t)+\tilde{\Sigma }(x,t)$ where $\tilde{\Sigma }(x,t)$ decays exponentially. The constant $\alpha _{T,\varepsilon }$ is positive and depends only on the initial condition (or, effectively, *T*) and $\varepsilon$.

This shows that

40 $$\begin{aligned} \alpha _{\varepsilon ,T}p_{1,\varepsilon }(x)-\Sigma _{T}(x,t)\le {}W_1(x,t)\le {}e^{a_{2,M}\eta ^{-1}e^{-\eta {}T}}\left( \alpha _{\varepsilon ,T}p_{1,\varepsilon }(x)+\Sigma _{T}(x,t)\right) , \end{aligned}$$

for $$(x,t)\in \mathbb {R}\times {}[T+\frac{\gamma }{\varepsilon },\infty )$$.

We first claim that $\alpha _{\varepsilon ,T}$ can be bounded above independently of *T*. To show that it is bounded, we take $T=0$ on the right-hand side of (40), and arbitrary *T* on the left. Sending $t\rightarrow \infty$ then implies that $\alpha _{\varepsilon ,T}p_{1,\varepsilon }(x)\le {}K_1\alpha _{\varepsilon ,0}p_{1,\varepsilon }(x)$ where $K_1$ is independent of $\varepsilon$ and *T*. Thus $\alpha _{\varepsilon ,T}\le {}K_1\alpha _{0,\varepsilon }$. This enables us also to find a subsequence $\alpha _{\varepsilon ,T_j}$ such that .

We can now argue by contradiction to show the lemma. We suppose there is no constant $\alpha _\varepsilon$ such that . In particular, there exists a constant $\nu >0$ and sequence of times $t_i$ and points $x_i$ such that $|W_1(x_i,t_i)-\alpha _{\varepsilon ,\infty }p_{1,\varepsilon }(x_i)|>\nu$. We can further assume $x_i$ are bounded, since $W_1(x,t)$ decays exponentially (from Lemmas 10 and 9) and otherwise we would have a contradiction with the preceding inequality. We can now pick a convergent subsequence (relabelling)  so that, because $W_1(x,t)$ is locally uniformly bounded,  and $|w_1-\alpha _{\varepsilon ,\infty }p_{1,\varepsilon }(z)|>\nu$. Without loss of generality, we let $\{T_j\}_{j}$ to be equal to $\{t_i\}_{i}$. Taking $x=x_j$, $t=t_i$, $T=t_i$ and sending $i\rightarrow \infty$ in (40) yields $w_1-\alpha _{\varepsilon ,\infty }p_{1,\varepsilon }(z)=0$, which contradicts $|w_1-\alpha _{\varepsilon ,\infty }p_{1,\varepsilon }(z)|>\nu >0$. $\square$

We note that we have not shown an exponential decay in time for $\Sigma (x,t)=W_1(x,t)-\alpha _{\varepsilon ,\infty }p_{1,\varepsilon }(x)$, unlike the situation in the proof of Lemma 5 and in Iglesias and Mirrahimi (2018, 2021). Nevertheless, we get immediately equivalent results to Lemmas 5 and 6 with no modifications to the proof.

#### Lemma 13

Under the assumptions $\lambda _{\underline{i},0}<0$, $\lambda _{1,0}\ne {}\lambda _{2,0}$, $\varepsilon$ is small enough, and (B1)-(B4) the normalized population will converge to $p_\varepsilon$ as $t\rightarrow \infty$, i.e.

#### Lemma 14

Under the assumptions $\lambda _{\underline{i},0}<0$, $\lambda _{1,0}\ne {}\lambda _{2,0}$, $\varepsilon$ is small enough, and (B1)-(B4) the total population $\rho _\varepsilon (t)$ will convergence to a finite, positive value

#### Proof of Theorem 3

Theorem 3 now follows by combining the preceding two lemmas with Theorem 1 for the case of a single peak. $\square$

## Numerical computations

To complement theoretical results, we present several numerical examples which illuminates interesting features of the transient dynamics, as well as demonstrating our conclusions for the long time behaviour.

### Description of methods

To obtain the numerical results, we make use of a simple finite difference scheme. We discretize space, which we take as [0, *L*] as $x_{i}={\Delta {x}}i$ for $i=1,\ldots N_{x}$ where $x_{N_{x}}=L$. We discretize the time interval [0, *T*] as $t_{i}={\Delta {t}}i$ for $i=1,\ldots ,N_{t}$ where ${\Delta {t}}N_{t}=T$. Iterations are computed using the forward-in-time Euler scheme with a centre difference approximation of the Laplacian:

$$\begin{aligned} n^{k+1}_{j}={\left\{ \begin{array}{ll} n^{k}_{j}+\frac{{\Delta {t}}}{{\Delta {x}}^2}(n^{k}_{j+1}-2n^{k}_{j}+n^{k}_{j-1})+{\Delta {t}}n^{k}_{j}a(x_{j},t_{k+1}) & \text { for } j=2,\ldots ,N_{x}-1,k=1,\ldots ,N_{t}\\ 0 & \text { for } j=1,N_{x},k=1,\ldots ,N_{t}\\ \end{array}\right. } \end{aligned}$$

Although we’re seeking to approximate a solution on an unbounded domain, we apply Dirichlet boundary conditions rather than, for instance, truncating the spatial domain at each time point, because as the solution is expected to concentrate we suppose that the boundary conditions ultimately do not significantly impact the final solution.

We also make use of an adaptation of the asymptotic preserving scheme given in Calvez et al. (2022). For a detailed description, we refer to their paper but will summarise the key points. This scheme works with the Hamilton-Jacobi equation

41 $$\begin{aligned} {\left\{ \begin{array}{ll} \partial _{\tau }\bar{u}_{\varepsilon }+\vert \partial _{x}\bar{u}_\varepsilon -\frac{c}{2}\vert ^2=\varepsilon \partial _{xx}\bar{u}_\varepsilon -\left( a(x)-\frac{c^2}{4}-\rho _\varepsilon (t)\right) \\ \rho _\varepsilon (t)=\int {}e^{-\frac{\bar{u}_\varepsilon }{\varepsilon }}dx \end{array}\right. } \end{aligned}$$

so that $N=e^{\frac{-\bar{u}_\varepsilon }{\varepsilon }}$ solves (5).

Where $\bar{u}^{0}$ is specified, the iterations are then given by

42 $$\begin{aligned} {\left\{ \begin{array}{ll} \frac{\bar{u}^{n+1}_{i}-\bar{u}^{n}_{i}}{\Delta {t}}+H\left( \frac{\bar{u}^{n}_{i}-\bar{u}^{n}_{i-1}}{\Delta {}x}-\frac{c}{2},\frac{\bar{u}^{n}_{i+1}-\bar{u}^{n}_{i}}{\Delta {}x^{{2}}}-\frac{c}{2}\right) =\varepsilon \frac{\bar{u}^{n}_{i+1}-2\bar{u}^{n}_{i}+\bar{u}^{n}_{i-1}}{{\Delta {x}}}-\left( a(x_{i})-\frac{c^2}{4}-\rho _{n}\right) \\ \rho _{n}={\Delta {x}}\sum _{i\in \mathbb {Z}}e^{-\frac{\bar{u}^{n}_{i}}{\varepsilon }}. \end{array}\right. } \end{aligned}$$

Here

$$\begin{aligned} H(p,q)=\max \{H^{+}(p),H^{-}(q)\} \end{aligned}$$

where

$$H^{+}(p)= {\left\{ \begin{array}{ll} p^2 \text { if } p>0,\\ 0 \text { if } p<0. \end{array}\right. }$$

and

$$H^{-}(q)= {\left\{ \begin{array}{ll} 0 \text { if } q>0,\\ q^2 \text { if } q<0. \end{array}\right. }$$

When $c=0$ this is exactly the scheme $S_\varepsilon$ in Calvez et al. (2022). We note that although the actual schemes are almost identical, we have quite different assumptions on the growth term, which is for us given by $R(x,I)=a(x)-\frac{c^2}{4}-I$. In particular, for $I=0$ this is negative for $|x|>R_{0}$ but in Calvez et al. (2022) there exists an $I_{m}>0$ such that $R(x,I_{m})>0$ for all *x*. We find that the scheme converges to what is expected but to prove the convergence is beyond the scope of the current paper and we leave it for future work.

Associated with the above scheme is also a limit scheme

43 $$\begin{aligned} {\left\{ \begin{array}{ll} \frac{v^{n+1}_{i}-v^{n}_{i}}{\Delta {t}}+H\left( \frac{v^{n}_{i}-v^{n}_{i-1}}{\Delta {}x}-\frac{c}{2},\frac{v^{n}_{i+1}-v^{n}_{i}}{\Delta {}x}-\frac{c}{2}\right) =-\left( a(x_{i})-\frac{c^2}{4}-P_{n}\right) \\ \min _{i\in \mathbb {Z}}v_{i}^{n+1}=0. \end{array}\right. } \end{aligned}$$

Here $P_{n+1}$ is the limiting value of $\rho _{n+1}$ as $\varepsilon \rightarrow {0}$ which is unique according to Calvez et al. (2022). This is computed by finding the root of the following function

$$\begin{aligned} J \mapsto \min _{i\in \mathbb {Z}}\left\{ v^{n+1}_{i}-\Delta {t}H\left( \frac{v^{n}_{i}-v^{n}_{i-1}}{\Delta x}-\frac{c}{2},\frac{v^{n}_{i+1}-v^{n}_{i}}{\Delta x}-\frac{c}{2}\right) -\Delta {t}R(x_{i},J)\right\} . \end{aligned}$$

This function’s unique root is $P_{n+1}$ according to Remark 4.3 in Calvez et al. (2022).

### Results

The following numerical simulations provide additional support for our conclusions about the long time behaviour of solutions. Moreover, they also provide insights into the transient behaviour which is not captured by the theorems in Section 3.

**Fig. 4:**
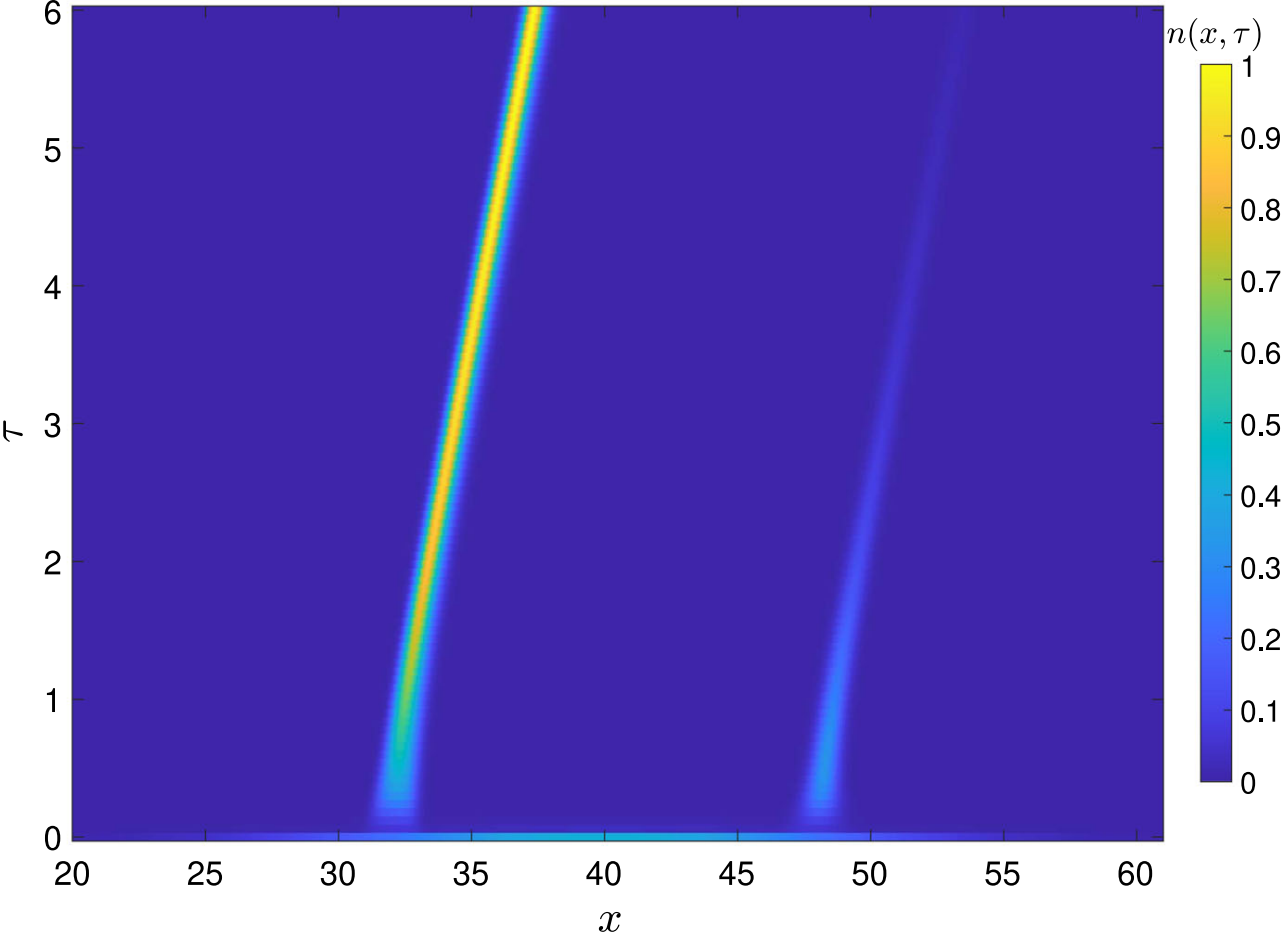
Dynamics of $n(x,\tau )$ on a fixed space and time interval. The initial condition is taken as a $n_{0}(x)=\frac{1}{10}e^{-\frac{(x-37.5)^2}{10^2}}$. We choose ${d}=\frac{1}{2}$, $a_{1}(x)=(\frac{5}{2}-(x-35)^4)^{+}$, $a_{2}=\left( \frac{5}{2}-(x-40)^2\right) ^{+}$, $\varepsilon =0.1$ and $c_{1}=c_{2}=1$

In Fig. 4 we plot the solution $n(x,\frac{t}{\varepsilon })$. We see that the solution does in fact concentrate, as proved in Theorem 2, on the lagged optimum following the global optimum which satisfies $x_{i}=\text {argmin}_{z\in \{x_{1},x_{2}\}}|a_{i}''(z)|$. This is not captured by Theorem 2 although it is suggested by it.

**Fig. 5:**
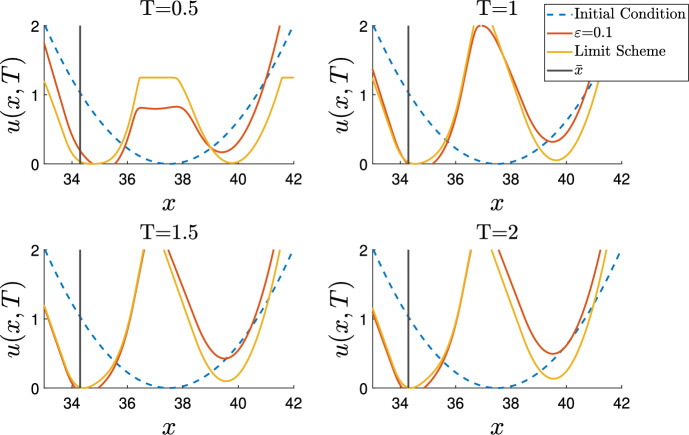
The solution *u*(*x*, *T*) to (42) and (43) for $T\in \{\frac{1}{2},1,\frac{3}{2},2\}$. The initial condition is taken as $u_{\varepsilon }(x,0)=-\varepsilon \left( \log (\frac{1}{10})-\frac{(x-37.5)^2}{10^2}\right)$. We choose ${d}=\frac{1}{2}$, $a_{1}(x)=(\frac{5}{2}-(x-35)^4)^{+}$, $a_{2}=\left( \frac{5}{2}-(x-40)^2\right) ^{+}$, $\varepsilon =0.1$ and $c_{1}=c_{2}=1$

We can compare results using the other numerical scheme too. In Fig. 5, we plot $\bar{u}$ which is the numerical solution approximating $u_\varepsilon =-\varepsilon \log {N}$ at four time points. Since the solution is time-dependent, there is a time-dependent minimum *x*(*T*) where $u(x_{T},T)=0$. We find that, as expected, *x*(*T*) approaches the $\bar{x}_{1}$ both for the $\varepsilon >0$ scheme and the limit scheme.

Moreover, the results show that the solution initially concentrates near both peaks, even though ultimately one dies out. This suggests that both subpopulations (following $x_{1}$ and $x_{2}$) will coexist for some significant time (recall the time units are $\tau =\frac{t}{\varepsilon }$).

We can also compare this to results for an asymptotic scheme which we adapt from Calvez et al. (2022) in Fig. 5. We observe that the limiting scheme and the $\varepsilon >0$ scheme both reproduce the observation of the finite differences method where the solution concentrates only at the lagged optimum $\bar{x}_{i}$ associated to peak with minimal $|a''(x_{i})|$.

**Fig. 6:**
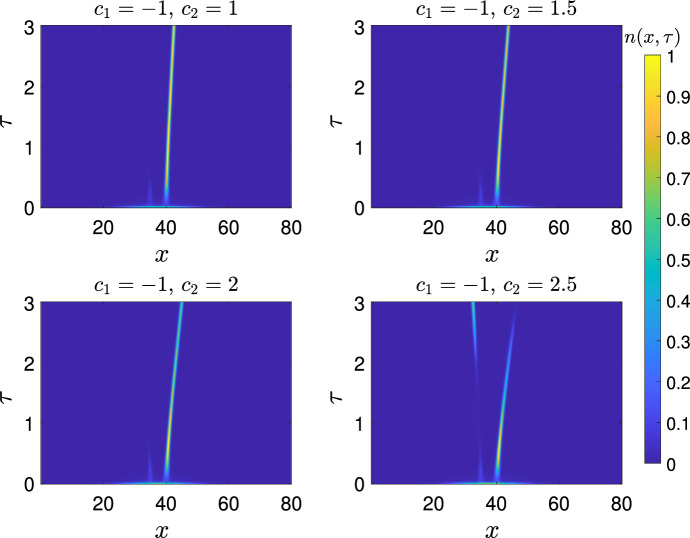
We choose ${d}=-\frac{1}{2}$, $a_{1}(x)=(\frac{7}{4}-(x-32)^2)^{+}$, $a_{2}=\left( \frac{5}{2}-(x-48)^2\right) ^{+}$, $\varepsilon =0.1$ and $c_{1}=-1$ and $c_{2}$ to vary as in the above plots

Next, we investigated the effect of increasing the speed $c_{2}$ while leaving $c_{1}$ fixed. As shown in in Fig. 6, when $c_{2}$ is small, $a_{1,M}-\frac{c_{1}^2}{4}<a_{2,M}-\frac{c_{2}^2}{4}$ and so we expect the solution to concentrate on $\bar{x}_{2}+\varepsilon {}c_{2}t$ in the long time, as required by Theorem 3. However when $c_{2}$ is large enough we will instead have $a_{1,M}-\frac{c_{1}^2}{4}>\max \left\{ {d},a_{2,M}-\frac{c_{2}^2}{4}\right\}$ and we expect concentration at $\bar{x}_{1}+\varepsilon {}c_{1}t$ in the long time. Indeed, this occurs, and we also see that for $c_{2}=2.5$ initially the subpopulation following $\bar{x}_{2}+\varepsilon {}c_{2}t$ grows and the subpopulation following $\bar{x}_{1}+\varepsilon {}c_{1}t$ decays. We expect that this is due to the fact that there is initially mass near the true optimum at $x_{2}$ and $a_{2,M}>a_{1,M}$. This allows an initially higher growth rate near ${x}_{2}+\varepsilon {}c_{2}t$ for small times. The competition then suppresses the growth everywhere else. However, due to the shift, the population near ${x}_{2}+\varepsilon {}c_{2}t$ cannot be sustained and starts to lag, allowing the population near $\bar{x}_{1}+\varepsilon {}c_{1}t$, which has the higher lagged fitness, to overtake.

We also looked at the effect of initial conditions on the transient behaviour. We keep the parameters fixed and find that the initial conditions can alter where the solution initially concentrates. In particular, for nearby initial conditions, it will concentrate on some point which is likely the single lagged optimum of $a_{1}(x-\varepsilon {}c_{1}t)+a_{2}(x-\varepsilon {}c_{2}t)$ before the sufficiently separate and it instead follows the maximum of the positive lagged optima.

Finally, we determine the behaviour of $\rho _\varepsilon (t)=\int _{\mathbb {R}}n(x,t)dx$, or the total population in Fig. 8 for an example initial condition where the two peaks overlap. In this case, $\rho _\varepsilon (t)$ is non-linear but eventually monotonic. If this property could be established rigorously, it would simplify some of the proofs presented here, in particular Theorem 3.7

**Fig. 7:**
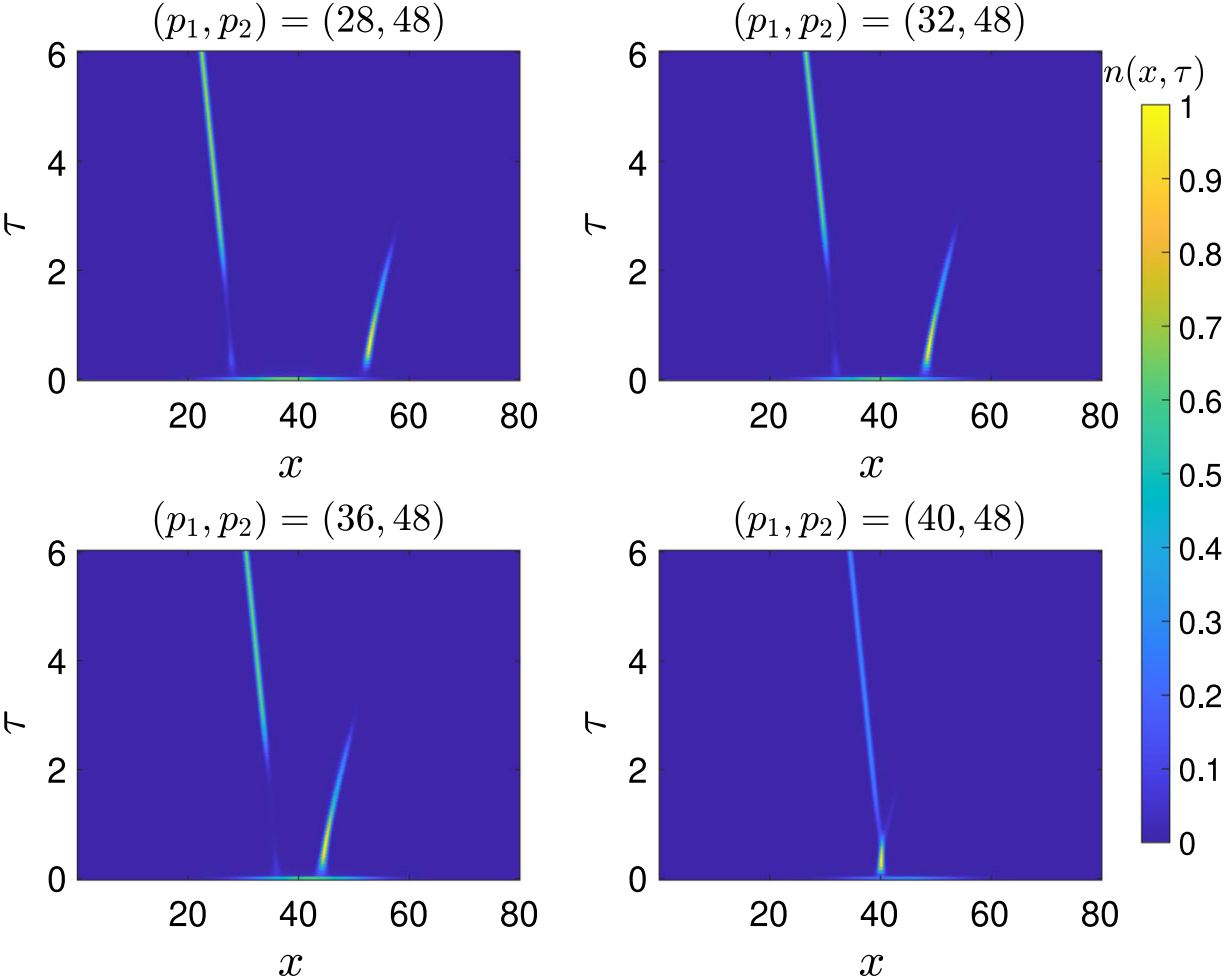
The long time behaviour of $n(x,\tau )$ for a range of initial conditions. We choose ${d}=-\frac{1}{2}$, $a_{1}(x)=(\frac{7}{4}-(x-p_{1})^2)^{+}$, $a_{2}=\left( \frac{5}{2}-(x-p_{2})^2\right) ^{+}$, $\varepsilon =0.1$ and $c_{1}=-1$ and $c_{2}=2.5$. We pick $(p_{1},p_{2})=(28+z,52-z)$ for $z=12,8,4,0$

We find that for such an initial condition solution concentrates at a point which is initially at neither $\bar{x}_{i}+\varepsilon {}c_{i}t$ due to the overlapping support of $a_{1}$ and $a_{2}$. Once these have separated sufficiently, it concentrates at the lagged optima which has the maximum fitness.

**Fig. 8:**
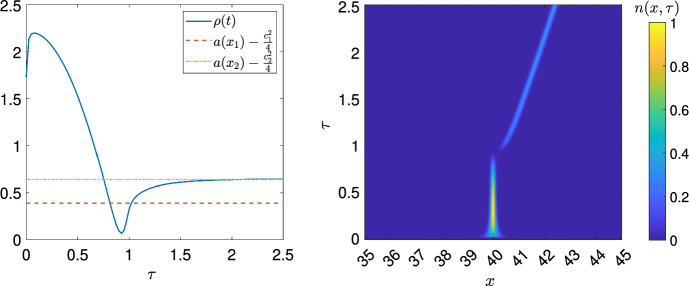
Dynamics of $\rho (\tau )$ and corresponding dynamics of $n(x,\tau )$. We choose ${d}=\frac{1}{2}$, $a_{1}(x)=(\frac{7}{4}-(x-40)^2)^{+}$, $a_{2}=\left( \frac{5}{2}-(x-40)^2\right) ^{+}$, $\varepsilon =0.05$ and $c_{1}=-\frac{6}{5}$ and $c_{2}=\frac{6}{5}$

## Discussion and future work

### Discussion

Our work has focused on understanding the long time behaviour of solutions to a novel integro-differential model of asexual reproduction in a temporally changing environment. Our model is novel in that it allows for locally optimal traits to move at different rates. We build on the work in Lorenzi and Pouchol (2020) which have a static fitness function with multiple global optima and in Iglesias and Mirrahimi (2021) which considers a linearly-shifting fitness function.

Specifically, we have answered the following questions in Theorems 1 to 3:

1. For which conditions on the shifting rate and intrinsic growth rates does the entire population go extinct?
2. Which trait/s dominate in long time, if the population does not go extinct?

Theorem 1, which applies to Case 1 where there are multiple global optima shifting with the same speed, shows that the solution concentrates on a subset of the "lagged optima" in the rare mutation and long time limit. Theorem 2 states the weighted rescalling will concentrate on only the shallowest optima. This, combined with the numerical results, suggests that it concentrates on the lagged optima behind the shallowest peaks. In Case 2, where there are multiple optima shifting at different speeds, Theorem 3 shows that if $a_{1,M}-\frac{c_{1}^2}{4}>\max \{a_{2,M}-\frac{c_{2}^2}{4},{d}\}$ then the solution concentrates on $\bar{x}_{1}$, and if the lagged fitness associated to both $a_{i}$ are negative, then the population goes extinct. In other words, if it does not go extinct, the solution concentrates on lagged optima with the largest fitness.

This shows that the dominant subpopulation depends on the lagged optimum fitness and not just the true optimal fitness. From the numerical results in Section 4, we observe that it can first appear that one subpopulation (the one following the optimum with the true optimal fitness) is dominant only to later be overtaken by the subpopulation with the higher lagged optimum. This is particularly relevant when the population initially begins with the optimal traits overlapping. This would be an interesting feature to look out for in cell populations in an ageing environment where there are known to be several evolutionary strategies to cope with the environmental change, i.e. the case of decoy fitness peaks mentioned in the introduction. Such a mechanism may be responsible for a sudden emergence of cancer cells, not because they are truly more suited to the environment, but because they can adapt more easily to a changing environment.

### Limitations and Future Work

Firstly, we remark that the Hamilton-Jacobi method is not the only one which can describe the evolution of a population such as this. An alternative approach is to work with the moments of the phenotypic distribution thereby reducing to the model to a system of ODEs which describe the evolution of, especially, the mean-trait, population size, and variance (Villa 2024; Guerand et al. 2023). In Villa (2024) the (infinite) system of ODEs for the moments of the phenotypic distribution are derived for a similar class of equations to (1), from which a closed system may obtained by truncation (i.e. neglecting moments above a certain order). The truncated system is more tractable analytically and computationally, however it remains an open question to determine the appropriate level of truncation and control the errors the truncation introduces. In Guerand et al. (2023), for the case of a sexually reproducing population in a static environment, it is shown that moment equations may be asymptotically closed for small mutation rates (segregational variance). The asymptotic closure relies on a dissipation term that arises due to the particular reproduction kernel considered hence this method is not applicable to our problem. It remains an open problem to determine when moment closures could apply to models such as (1).

With regards the specific modelling choices, we have made simplifications that allow for clearer presentation without sacrificing the generality of the results. In Case 2, by taking $a_{i}$ with compact support and constant *d* we are considering only models which have a *constant* negative fitness away from the optimal traits, whereas the previous model only required that the fitness be bounded above by a negative constant. Ultimately, this should not affect the main conclusions since the solutions localise to the moving lagged optima in the limits we will consider. Another simplification is that we choose to deal with only two $a_{i}$, each with a unique maximum, but the theory here is applicable to the case of any finite number of $a_{i}$, each with a finite number of maxima. Again, this is because the peaks all separate eventually so only interact through the competition term.

A challenging aim for future work is to extend the results here to higher dimensional trait space which is more relevant when there is competition between more than two traits. It seems plausible that one could determine which peak has the greatest local growth rate and thus reduce the problem to the relevant shifting one peak problem. In the higher dimensional case, however, we no longer have the explicit representation formula for the solutions to the limiting Hamilton-Jacobi equation, and further work would be required to show the concentration of the solution.

## Summary of previous results

### Related models

The problem where $a(x,t)=a(x)$ is time independent and has multiple optima$\text {argmax}_{x\in \mathbb {R}}{a(x)}=\{x_1,\ldots ,x_n\}$ in a bounded domain with Neumann boundary conditions is studied in Lorenzi and Pouchol (2020). This problem is given by

44 $$\begin{aligned} {\left\{ \begin{array}{ll} \partial _{t}n-\sigma \partial _{xx}n=n\left( a(x)-\int _{\mathbb {R}}n(y,t)dy\right) , & (x,t)\in {\Omega }\times {}\mathbb {R}^{+}, \\ n(x,0)=n_{0}(x), & \\ \nabla {n}\cdot \nu (x)=0, & (x,t)\in {\partial {\Omega }}\times {}\mathbb {R}^{+},\ \end{array}\right. } \end{aligned}$$

where $\nu (x)$ is the outward unit normal. For problems in smooth bounded domains, or for periodic parabolic problems (even in unbounded domains), one can use the Krein-Rutnman theorem to assert the existence of a principal eigenvalue and associated positive eigenfunction solution for the linearised problem. The Krein-Rutman theorem is a generalisation of the well-known Perron–Frobenius theorem (which asserts the existence of a positive eigenvalue and eigenvector for square matrices where all entries are positive) to positive operators on Banach spaces (Lam and Lou 2022). This is useful because one can usually relate the solutions for arbitrary initial conditions to the solution to the eigenvalue problem which is easier to analyse.

The problem without periodic coefficients and in an unbounded domain excludes the use of the Krein-Rutman theorem. According to Berestycki and Rossi (2015): "The Krein-Rutman theory cannot be applied if $\Omega$ is nonsmooth or unbounded (except for problems in periodic settings), because the resolvent of *L* [the operator in question] is not compact." Our problem (2) must be considered in the unbounded domain due to the term $a(x-\tilde{c}t)$. By making a coordinate change to $z=x-\tilde{c}t$ and performing a Liouville transform we can reduce (2) to almost exactly the problem (44) except for the fact the domain is unbounded and we need to be able to carrying over results from the transformed problems to the original. In particular we cannot simply undo the Liouville transform once we find the limiting solution as $\varepsilon \rightarrow {0}$ since it depends on $\varepsilon$.

It is shown that the solution to the problem on a bounded domain (44) with $\sigma =\varepsilon$ converges as $t\rightarrow \infty$ to a multiple of the solution to the eigenvalue problem

45 $$\begin{aligned} {\left\{ \begin{array}{ll} -\varepsilon \partial _{xx}\psi _\varepsilon -a(x)\psi _\varepsilon =\lambda _\varepsilon \psi _\varepsilon & x\in {\Omega }, \\ \nabla {\psi }\cdot \nu (x)=0 & x\in {\partial {\Omega }},\\ \psi \ge {0} & x\in \Omega . \end{array}\right. } \end{aligned}$$

Furthermore, . From these two facts, one can conclude the solution concentrates on some subset of the maximum points of *a*(*x*). Due to this concentration result, can be expected that the fact that the domain is unbounded should not matter (i.e. the highly concentrated solutions should be relatively unaffected by the boundary conditions at any fixed distance as $\varepsilon \rightarrow {0}$).

To establish the existence of a solution $\psi _\varepsilon$ to (45), and also its convergence to a sum of Dirac deltas, one makes use of Krein-Rutman theorem which gives a variational formula of the eigenvalue (also known as the Rayleigh-Quotient formula).

#### Lemma 15

(Lemma 1 in Lorenzi and Pouchol (2020)) There exists an eigenvector, eigenvalue pair $(\psi _\varepsilon ,\lambda _\varepsilon )$ solving (45). The eigenfunction is unique up to normalization and the eigenvalue is characterised by the following formula:

$$\begin{aligned} \lambda _\varepsilon =\inf _{\phi \in H^{1}(\Omega )\backslash \{0\}}\frac{\varepsilon \int _{\Omega }|\nabla \phi |^2-\int _{\Omega }a(x)\phi ^2}{\int _{\Omega }\phi ^2}. \end{aligned}$$

Using this lemma, one gets that $\psi _\varepsilon$ concentrates by: firstly, choosing an appropriate sequence $\psi _\varepsilon$ such that $\lambda _\varepsilon \rightarrow {-a_{M}}$. Then a simple computation shows, for a test function $\eta \in {C(\overline{\Omega })}$ supported away from $\{x_{1},\ldots x_{n}\}$, that

Unfortunately, the use of the Rayleigh-Quotient is lost for unbounded domains except under some specific conditions on the fitness function *a*(*x*), for instance, if the fitness function is confining ($\lim _{|x|\rightarrow \infty }a(x)=-\infty$). The condition comes from the study of confining potentials in quantum mechanics, and the authors of Alfaro and Veruete (2019) obtain some results regarding evolutionary branching under the assumption the fitness function is a confining potential.

After establishing the basic concentration results, the authors of Lorenzi and Pouchol (2020) are able to find further constraints for the subset of $\{x_{1},\ldots ,x_{n}\}$ where the solution eventually concentrates: their Proposition 2 shows an example of a symmetric fitness function *a*(*x*) which leads to equal concentration on two peaks, and their Proposition 3 refines the concentration set according the concavity of *a*(*x*) at each of its maxima. They borrow this result from semi-classical analysis, as given in Holcman and Kupka (2006) for operators defined on a compact Riemannian manifold, independent of boundary conditions. We will usually consider Dirichlet boundary conditions when working with approximate problems on bounded domains, of the general form:

46 $$\begin{aligned} {\left\{ \begin{array}{ll} -\varepsilon \partial _{xx}\psi _\varepsilon -a(x)\psi _\varepsilon =\lambda _\varepsilon \psi _\varepsilon & x\in {\Omega }, \\ {\psi }=0 & x\in {\partial {\Omega }},\\ \psi \ge {0} & x\in \Omega . \end{array}\right. } \end{aligned}$$

We borrow the same result, phrased suitably, for our problem.

#### Lemma 16

(Proposition 3 in Lorenzi and Pouchol (2020)) Let $S(x)=|a''(x)|$ for $x\in {}M:=\{x_{1},\ldots ,x_{n}\}=\text {argmax}_{x\in \Omega }a(x)$. Let $M_{1}=\text {argmin}_{x_{j}}S(x_{j})$. Then the solution $\psi _\varepsilon$ to (46), satisfies, up to extraction of subsequences:

Although in Lorenzi and Pouchol (2020) this result holds for a Neumann problem, the semiclassical analysis result they use remains applicable to Dirichlet problems.

We will analyse and find the concentration results for the problem (2) for the scaling $\tilde{c}=c\varepsilon$. We aim to do this by similarly analysing the eigenvalue problem, using the results from Húska and Polacik (2008) which construct solutions to the eigenvalue problem for such parabolic problems on unbounded domains as a limit of Dirichlet problems on bounded domains.

The authors of Iglesias and Mirrahimi (2021) (who take $a(x,t)=a(e(t),x-\tilde{c}t)$ for some periodic function *e*(*t*) with period *T*) also make use of theory presented in Húska and Polacik (2008). Using the scaling $\tilde{c}=c\varepsilon$ they show that the shifted solution $N_\varepsilon (x,t):=n_{\varepsilon }(x+c\varepsilon {}t,t)$ will concentrate at a point $\bar{x}$ which they call the lagged optima. Letting $\bar{a}(x)=\frac{1}{T}\int _{0}^{T}a(e(t),x)$ and $x_{m}$ be the unique maxima of $\bar{a}$, the authors show that the lagged optima satisfies the following equation

$$\begin{aligned} \bar{a}(\bar{x})=a(x_{m})-\frac{\tilde{c}^2}{4\sigma }. \end{aligned}$$

In particular, if the right-hand side is negative, the population dies out.

We are able to show that the solution $M_{\varepsilon }(x,t)$ to (7) concentrates on some set of finite points as $\varepsilon \rightarrow {0}$ by using theory presented (Húska and Polacik 2008) to reduce the problem to one in a finite domain, then combining the results in Lorenzi and Pouchol (2020) and Iglesias and Mirrahimi (2021) to determine these locations more precisely, and to show under what conditions we can obtain concentration to a single location.

It is beyond the scope this paper to translate this result to what it means for $n_\varepsilon (x,t)$ the solution to (2). We predict that the Liouville transform will have simply shifted these concentration points but a precise characterisation of $\varepsilon \log {}(n_\varepsilon )$ would be required to find the locations. This is exactly what one studies when performing a WKB-transform, but for this particular problem difficulties are encountered in determining uniqueness of this solution.

### Generalised super- and sub- solutions

It is necessary to glue together and construct potentially non-smooth super- and sub- solutions throughout this paper. To this end, we review the notion of generalised sub- and super- solutions as discussed in Lam and Lou (2022) Chapter 1, Section 1, and we paraphrase the Definition 1.1.1 here. Let $\Omega \subset \mathbb {R}^N$ be a possibly unbounded domain, define $\Omega _T=\Omega \times {}(0,T]$. Consider the following operator (using index notation):

$$\begin{aligned} \mathcal {L}u:=-a^{ij}\partial _{ij}u-b^{i}\partial _{i}u-cu. \end{aligned}$$

We suppose that $a^{ij},b^{i},c\in {}C^{0}(\overline{\Omega }_T)$, and that $a^{ij}$ is uniformly elliptic, that is there is a $\lambda _0>0$ such that $\lambda _0{}|\zeta |^2\le {}a^{ij}(x,t)\zeta _i\zeta _j$ for all $\zeta \in \mathbb {R}^N$ and $(x,t)\in \Omega _T$.

#### Definition 1

A function $\underline{u}\in {}C(\Omega _{T})$ satisfies the inequality

$$\begin{aligned} \partial _{t}\underline{u}-\mathcal {L}\underline{u}\le f(x,t,\underline{u},D\underline{u}), \end{aligned}$$

in the generalised sense if for every $(x_0,t_0)\in \Omega _T$ there exists a neighbourhood *U* of $(x_0,t_0)$ and a function $\tilde{u}\in {}C^{2,1}(\overline{U})$ such that $\tilde{u}\le {}\underline{u}$ in *U*, $\underline{u}(x_0,t_0)=\tilde{u}(x_0,t_0)$, and

$$\begin{aligned} \partial _{t}\tilde{u}(x_0,t_0)-\mathcal {L}\tilde{u}(x_0,t_0)\le f(x_0,t_0,\tilde{u}(x_0,t_0),D\tilde{u}(x_0,t_0)). \end{aligned}$$

Then $\underline{u}$ is called a generalised subsolution.

One obtains the following comparison theorem, adapted to unbounded domains (Lam and Lou 2022, Chapter 6, Section 6.2, Theorem 6.2.1)

#### Theorem 4

Suppose *u* is a generalised subsolution of

$${\left\{ \begin{array}{ll} \partial _{t}u-\mathcal {L}u\le 0~~(x,t)\in \Omega \times (0,T),\\ u(x,t)\le 0~~(x,t)\in \partial \Omega \times (0,T),\\ u(x,0)\le 0, \end{array}\right. }$$

which satisfies

$$\begin{aligned} \liminf _{R\rightarrow \infty }e^{-kR^2}\left[ \max _{\begin{array}{c} x\in \Omega ,|x|=R,\\ 0\le t\le {T} \end{array}}u(x,t)\right] \le 0, \end{aligned}$$

then $$u\le {}0$$ in $$\Omega \times (0,T)$$.

We will make frequent use of this direct consequence

#### Theorem 5

Suppose *u* is a solution of

$${\left\{ \begin{array}{ll} \partial _{t}u-\mathcal {L}u=0~~(x,t)\in \mathbb {R}\times (0,T),\\ u(x,0)=u_0(x), \end{array}\right. }$$

and that *v* is a generalised subsolution

$${\left\{ \begin{array}{ll} \partial _{t}v-\mathcal {L}v\le 0~~(x,t)\in \mathbb {R}\times (0,T),\\ u(x,0)=v_0(x). \end{array}\right. }$$

If $v_0(x)-u_0(x)\le {0}$ and $w=v(x,t)-u(x,t)$ satisfies

$$\begin{aligned} \liminf _{R\rightarrow \infty }e^{-kR^2}\left[ \max _{\begin{array}{c} x\in \Omega ,|x|=R,\\ 0\le t\le {T} \end{array}}w(x,t)\right] \le 0, \end{aligned}$$

then $$v(x,t)\le {}u(x,t)$$ for $$(x,t)\in \mathbb {R}\times (0,T)$$.

The analogous theorems for generalised supersolutions are obtained by reversing the inequalities.

### Principal Floquet Bundles for Linear Parabolic Equations with Time Dependent Coefficients

This reviews the main result we need from Húska and Polacik (2008) and is included in the introduction for completeness.

Consider the following two problems. First, on the whole space $\mathbb {R}^N$

47 $$\begin{aligned} \partial _{t}u={\Delta {u}+\underline{b}\cdot {}\nabla {u}}+A(x,t)u \text { on } \mathbb {R}^N\times {(s,\infty )} \end{aligned}$$

where *s* is some arbitrary number in $\mathbb {R}$, $\underline{b}\in \mathbb {R}^n$, and $A(x,t)\in {}L^\infty (\mathbb {R}^N,\mathbb {R})$. Secondly on a ball of radius *R*, the Dirichlet problem:

48 $$\begin{aligned} {\left\{ \begin{array}{ll} \partial _{t}u={\Delta {u}+\underline{b}\cdot {}\nabla {u}}+A(x,t)u & (x,t)\in {}B_{R}\times {(s,\infty )},\\ u\ge {0}, & \\ u=0 & (x,t)\in {}\partial {}B_{R}\times {(s,\infty )} \end{array}\right. } \end{aligned}$$

In Húska and Polacik (2008), the subsequent results are initially obtained in the case where $\underline{b}=\underline{0}$, however these are then developed for operators yet more general than what we consider here (see their Section 9).

Following Húska and Polacik (2008), we require the following assumptions. Note that all constants are positive.

1. There are constants $A_{0}$ and $r_{0}$ such that $\Vert {}A\Vert _{L^{\infty }(\mathbb {R}^N\times {\mathbb {R}})}\le {}A_{0}$, and $A(x,t)\le {0}$ a.e for $|x|\ge {r_{0}}$.
2. For each $s=s_{0}$ there is a solution $\phi$ of (47) such that $\phi (.,t)\in {L^\infty (\mathbb {R}^N)}$ for all $t\ge {}s_{0}$ and for some positive constants $\varepsilon$ and *C*, we have:
  $$\begin{aligned} \frac{\Vert \phi (.,t)\Vert _{L^\infty (\mathbb {R}^N)}}{\Vert \phi (.,s)\Vert _{L^\infty (\mathbb {R}^N)}}\ge Ce^{\varepsilon (t-s)} \text { for } s_{0}\le {s}\le {t}. \end{aligned}$$

As is proved in Húska and Polacik (2008) this is equivalent to the hypothesis on (48), which is that

(C3) There are constants $R_{0}$, $C_{0}$ and $\varepsilon _{0}$ such that for each $s=s_{0}$ the problem (48) with $R=R_{0}$ has a positive solution $u(.,s_{0})\in {}L^\infty (\mathbb {R}^N)$ and
  $$\begin{aligned} \frac{\Vert {u}(.,t)\Vert _{L^\infty (\mathbb {R}^N)}}{\Vert {u}(.,s)\Vert _{L^\infty (\mathbb {R}^N)}}\ge C_{0}e^{\varepsilon _{0}(t-s)} \text { for } s_{0}\le {s}\le {t}. \end{aligned}$$

The latter will be easier to show in general, and as remarked in Húska and Polacik (2008), the second hypothesis holds for all $R>R_{0}$ if it is shown to hold for $R=R_{0}$.

To state the first theorem we will later use, we need to also introduce the adjoint problem to (47)

49 $$\begin{aligned} -\partial _{t}v-\Delta {}v=A(x,t)v \text { in } \mathbb {R}^N\times {(s,\infty )}. \end{aligned}$$

This is obtained from (48) by the change of variables $t\rightarrow {-t}$.

We have the following theorem:

#### Theorem 6

(From Theorems 2.1 and 2.2 in Húska and Polacik (2008)) There exist positive solutions $\phi$ of (47) and $\psi$ of the adjoint problem (49), both with $s=-\infty$.

Let

$$\begin{aligned} X_{1}(t)=\text {span}\{\phi (.,t)\} \end{aligned}$$

and

$$\begin{aligned} X_{2}(t)=\{v\in L^\infty (\mathbb {R}^N):\int _{\mathbb {R}^N}\psi (x,t){v(x)}dx=0\}. \end{aligned}$$

The following are true

(i) $$X_{1}(t)\oplus {}X_{2}(t)=L^{\infty }(\mathbb {R}^N)$$ for all $$t\in \mathbb {R}$$.
(ii) $X_{1}$ and $X_{2}$ are invariant. That is, if $u(.,t;s,u_{0})$ is a solution to (47) with initial condition $u_{0}\in {}X_{i}(s)$ then $u(.,r;s,u_{0})\in {}X_{i}(t)$ for all $t\ge {s}$.
(iii) There are positive constants *C* and $\gamma$ such that for any $u_0\in {}X_{2}(s)$, we have
  $$\begin{aligned} \frac{\Vert {u(.,t;s,u_{0})}\Vert _{L^\infty (\mathbb {R}^N)}}{\Vert {\phi (.,t)}\Vert _{L^\infty (\mathbb {R}^N)}}\le Ce^{-\gamma (t-s)}\frac{\Vert {u_{0}}\Vert _{L^\infty (\mathbb {R}^N)}}{\Vert {\phi (.,s)}\Vert _{L^\infty (\mathbb {R}^N)}}~~(t\ge {s})~~. \end{aligned}$$

We also state a second lemma which is proved in Húska and Polacik (2008) during the course of proving the above theorem, but not presented as an independent result.

#### Lemma 17

A subsequence of solutions $\phi _{R_{n}}$ (48) will converge (as $n\rightarrow \infty$) locally uniformly in $\mathbb {R}^N\times [-t,t]$ for all $t>0$ to a solution $\phi$ of (47). Hence a positive entire solution to (47) exists.

We will mainly be interested in the following corollary of this theorem, which provides the long time behaviour for any (sensible) initial condition:

#### Corollary 2

Given any initial condition $u_{0}\in {}L^{\infty }(\mathbb {R}^N),$ there exists a constant $\alpha$ such that

This is a consequence of noting that we can decompose the initial condition as $u_{0}=\alpha \phi +v$ where $v\in {X_{2}(s)}$. If $u_0\notin {}X_2(0)$, for instance if $u_0\ge {0}$, then $\alpha$ is also non-zero. If $u(x,t)\ge {0}$ for all $t>0$ then $\alpha >0$.

## Proofs of technical lemmas

### Proof of Lemma 7

Here we prove Lemma 7 by providing some uniform bounds on $\psi _\varepsilon$. For the analogous eigenvalue problem in Iglesias and Mirrahimi (2021) it is remarked that this can be done but since the computations are similar they do not provide a proof, hence we do for completeness.

#### Proof

We recall that we may write

$$\begin{aligned} p_\varepsilon (x)=e^{\frac{\psi _\varepsilon (x)}{\varepsilon }}, \end{aligned}$$

so that $\psi _\varepsilon$ then solves:

50 $$\begin{aligned} -\varepsilon |\partial _{xx}\psi _\varepsilon |-\left| \partial _{x}\psi _\varepsilon -\frac{c}{2}\right| ^2-a(x)+\frac{c^2}{4}-\lambda _\varepsilon =0. \end{aligned}$$

Since $p_\varepsilon =A_\varepsilon {}p_{\varepsilon }^{\infty }$ where $A_\varepsilon =\Vert {p_\varepsilon ^{\infty }}\Vert _{L^{1}(\mathbb {R})}^{-1}$, the bounds from Lemma 3 imply

$$\begin{aligned} \varepsilon \log (A_\varepsilon )-\underline{\kappa }|x-x_\varepsilon |\le \psi _\varepsilon \le -\overline{\kappa }(|x|-R_0)+\varepsilon \log (A_\varepsilon ). \end{aligned}$$

Noting that the upper bound in Lemma 3 requires that $|x_\varepsilon |<R_0$, and that $A_\varepsilon <K$ for some constant $K>0$, we have

51 $$\begin{aligned} -K_2-\underline{\kappa }|x|<\psi _\varepsilon <-\overline{\kappa }|x|+K_2 \end{aligned}$$

This shows the locally uniform bounds.

We also need Lipschitz bounds. The Bernstein-type method presented in Iglesias and Mirrahimi (2021, 2018) can be used to obtain these. The idea is to show $\partial _{x}w_\varepsilon$ is a subsolution to some other elliptic PDE to which we can apply a maximum principal and obtain a uniform upper bound. First we define $w_\varepsilon =\sqrt{2K_2-\psi _\varepsilon }$ which solves:

$$\begin{aligned} -\varepsilon \partial _{xx}w_\varepsilon -\left( \frac{\varepsilon }{w_\varepsilon }-2w_\varepsilon \right) |\partial _{x}w_\varepsilon |^2-c\partial _{x}w_\varepsilon =\frac{a(x)+\lambda _\varepsilon }{-2w_\varepsilon }. \end{aligned}$$

Denote $W_{\varepsilon }=\partial _{x}w_\varepsilon$. We differentiate the above with respect to *x*, and multiply by $\frac{w_\varepsilon }{|w_\varepsilon |}$ to obtain:

$$\begin{aligned} -c\partial _{x}\vert W_\varepsilon \vert -\varepsilon \partial _{xx}\vert W_\varepsilon \vert -2\left( \frac{\varepsilon }{w_\varepsilon }-2w_\varepsilon \right) \partial _{x}\vert W_\varepsilon \vert W_\varepsilon +\left( \frac{\varepsilon }{w_\varepsilon ^2}+2\right) \vert W_\varepsilon \vert ^3 \end{aligned}$$

$$\begin{aligned} =\frac{a'(x)W_\varepsilon }{-2w_\varepsilon |W_\varepsilon |}+\frac{\vert W_\varepsilon \vert (a(x)+\lambda _\varepsilon )}{2w_\varepsilon ^2}. \end{aligned}$$

Firstly, using the bounds for $a(x),a'(x)$ and the following bound

$$\begin{aligned} \sqrt{K_2}\le w_\varepsilon \le \sqrt{K_3|x|}, \end{aligned}$$

where $K_3$ is a sufficiently large constant, we have the inequality

$$\begin{aligned} -c\partial _{x}\vert W_\varepsilon \vert -\varepsilon \partial _{xx}\vert W_\varepsilon \vert -2\left( K_4+K_5|x|^{\frac{1}{2}}\right) \vert W_\varepsilon \partial _{x}|W_\varepsilon |\vert +2\vert W_\varepsilon \vert ^3\le K_6+K_{7}|W_\varepsilon |, \end{aligned}$$

for positive constants $K_i$, $i=4,5,6,7$. This implies, for large enough $\Theta$ (depending on $K_i$) that

$$\begin{aligned} -c\partial _{x}\vert W_\varepsilon \vert -\varepsilon \partial _{xx}\vert W_\varepsilon \vert -2\left( K_4+K_{5}|x|^{\frac{1}{2}}\right) \vert W_\varepsilon \partial _{x}|W_\varepsilon |\vert +2(|W_\varepsilon \vert -\Theta )^3\le 0. \end{aligned}$$

We let $H(x)=\frac{R^2}{R^2-|x|^2}$ and $\overline{W}_{{R}}(x)=\Theta +{K_{8}}H(x)$ where $K_{8}$ is a constant to be chosen later. We show that $\overline{W}_{{R}}$ is a strict super solution of the preceding differential inequality, Since $\partial _{x}\overline{W}_{{R}}(x)={K_8}\frac{2x}{R^2}H(x)^2$ and $\partial _{xx}\overline{W}_{{R}}(x)={K_8}\frac{2}{R^2}H(x)^2+{K_8^2}\frac{8x^2}{R^{{4}}}H(x)^3$, see that, for $|x|<R$ we have

$$\begin{aligned}&-c\partial _{x}\overline{W}-\varepsilon \partial _{xx}\overline{W}-2(K_4+K_5|x|^{\frac{1}{2}})\vert \overline{W}\partial _{x}\overline{W}\vert +2(\overline{W}(x)-\Theta )^3\\&=K_8\left( -\frac{{2}cx}{R^2}H(x)^2-\frac{2\varepsilon }{R^2}H(x)^2-{K_8}\frac{8x^2\varepsilon }{R^{{4}}}H(x)^3\right) \\&\quad -2{K_8}(K_4+K_5|x|^\frac{1}{2})\frac{2|x|}{R^2}(H(x)^2\Theta +{K_8}H(x)^3)+2K_8^3H(x)^3\\&\ge {}\left( -K_8\frac{{2}c}{R}-\frac{2\varepsilon }{R^2}-{K_8}\frac{8\varepsilon }{R^2}-\frac{4{K_8}}{R}(K_4+K_5R^{\frac{1}{2}})(\Theta +{K_8})+2K_8^3\right) H(x)^3. \end{aligned}$$

Therefore, in order to show that the final expression is positive, we may choose $K_8$ sufficiently large depending on $K_i$ and *R*. We note that this choice can be made independent of *R* for $R>1$. We then have that, for $\varepsilon <1$,

$$\begin{aligned} -c\partial _{x}\overline{W}_{{R}}-\varepsilon \partial _{xx}\overline{W}_{{R}}-2\left( K_4+K_{5}|x|^{\frac{1}{2}}\right) \overline{W}_{{R}}|\partial _{x}\overline{W}_{{R}}|+2(\overline{W}_{{R}}-\Theta )^3>0. \end{aligned}$$

Since $|W_\varepsilon |\le \frac{1}{2\sqrt{K_2}}|\partial _{x}\psi _\varepsilon |$, and the bounds (51) imply $\psi _\varepsilon$ has at least one critical point, we have that there exists an $x^{*}$ such that $|W_\varepsilon |(x^{*})=0$. We may assume that $x^{*}=0$ by making the transformation $x\rightarrow {x+x^{*}}$. In this case, we replace *a*(*x*) with $\tilde{a}(x-x^{*})=a(x)$ in (50), which has all the same properties.

We now suppose that $\omega (x)=|W_\varepsilon |(x)-\overline{W}_{{R}}(x)$ attains a maximum at a point $z\in {}B_R({0})$, which is necessarily in the interior. If *R* is small enough, then the maximum $\alpha (z)$ is negative, since $W_\varepsilon$ is continuous and $\overline{W}>\Theta >0$. We therefore take *R* large enough that the maximum of $\alpha (x)$ in $B_R({0})$ is exactly 0. If such an *R* does not exist, we are done. If such an *R* does exist then, we have that $\partial _{x}\overline{W}(z)=\partial _{x}|W_\varepsilon |(z)$, $\overline{W}_{{R}}(z)=|W_\varepsilon |(z)$ and $-\partial _{xx}(|W|-\overline{W}_{{R}})(z)\ge {0}$. We deduce, using the above relations and differential inequalities satisfied by $|W_\varepsilon |$ and $\overline{W}_{{R}}$, that

$$\begin{aligned} 2(|W_\varepsilon |-\Theta )^3-2(\overline{W}_{{R}}-\Theta )^3<{0}, \end{aligned}$$

which is only possible if $|W_\varepsilon |(z)<\overline{W}_{{R}}(z)$ which contradicts $\overline{W}_{{R}}(z)=|W_\varepsilon |(z)$, and so no such *R* exists. Therefore $|W|(x)<\overline{W}_R(x)$ for all $R>1$. Taking the limit $R\rightarrow {\infty }$ we have that $|W_\varepsilon |(x)<\Theta +K_8$ for $x\in \mathbb {R}$, we have obtained the Lipschitz bound.

Given the $\varepsilon$-independent gradient bound on $\psi _\varepsilon$, and that it is therefore locally uniformly bounded, we use the Arzela-Ascoli theorem to conclude $\psi _\varepsilon$ converges locally uniformly (along subsequences) to a continuous function $\psi$. We now show that $\psi$ is indeed a viscosity solution. We check that for any test function *v* such that $\psi -v$ has a strict local maximum at $x_0$ we satisfy

$$\begin{aligned} -\left| \partial _xv(x_0)+\frac{c}{2}\right| ^2+a_M-a(x_0)\le 0, \end{aligned}$$

and for any test function *v* such that $\psi -v$ has a strict local minimum at $x_0$ we instead satisfy

$$\begin{aligned} -\left| \partial _xv(x_0)+\frac{c}{2}\right| ^2+a_M-a(x_0)\ge 0. \end{aligned}$$

If $\psi -v$ has a strict local maximum at $x_0$ then, because $\psi _{\varepsilon _j}$ converges to $\psi$ for some subsequence $\{\varepsilon _j\}_{j=1}^{\infty }$, it can be shown (see, for example, Evans 2022, Chapter 10) that for small enough $\varepsilon _j>0$ there is a point $x_j$ such that $\psi _{\varepsilon _j}-v$ has a strict maximum at $x_j$ and that . This gives us the relations $\partial _{x}\psi _{\varepsilon _j}(x_j)=\partial _{x}v(x_j)$ and $\partial _{xx}\psi _{\varepsilon _j}(x_j)\le {}\partial _{xx}v(x_j)$. We use these relations, and equation (50) to compute

$$\begin{aligned} \left| \partial _xv(x_j)+\frac{c}{2}{}\right| ^2+a_M-a(x)&= \left| \partial _{x}\psi _{\varepsilon _j}(x_j)+\frac{c}{2}\right| ^2+a_M-a(x_j)\\&=\varepsilon _j\partial _{xx}\psi _{\varepsilon _j}(x_j)+\lambda _{\varepsilon _j}+a_M-\frac{c^2}{4}\\&\le {}\varepsilon _j\partial _{xx}v(x_j)+\lambda _{\varepsilon _j}+a_M-\frac{c^2}{4}. \end{aligned}$$

Taking the limit as $j\rightarrow {\infty }$, and recalling that , yields the desires inequality that shows $\psi$ is a viscosity subsolution. Analogous computations apply to show it is a viscosity supersolution. We conclude that $\psi$ is a viscosity solution of (50). The constraint follows from the normalization. $\square$
